# Ameliorative effect of bofutsushosan (Fangfengtongshengsan) extract on the progression of aging-induced obesity

**DOI:** 10.1007/s11418-024-01803-4

**Published:** 2024-04-25

**Authors:** Takafumi Saeki, Saya Yamamoto, Junji Akaki, Takahiro Tanaka, Misaki Nakasone, Hidemasa Ikeda, Wei Wang, Makoto Inoue, Yoshiaki Manse, Kiyofumi Ninomiya, Toshio Morikawa

**Affiliations:** 1 Central R&D Laboratory, Kobayashi Pharmaceutical Co., Ltd, 1-30-3 Toyokawa, Ibaraki Osaka, 567-0057 Japan; 2 Pharmaceutical Research and Technology Institute, Kindai University, 3-4-1 Kowakae, Higashi-osaka, Osaka, 577-8502 Japan; 3 Laboratory of Medicinal Resources, School of Pharmacy, Aichi Gakuin University, 1-100 Kusumoto-cho, Chikusa-ku Nagoya, 464-8650 Japan; 4 Antiaging Center, Kindai University, 3-4-1 Kowakae, Higashi-osaka, Osaka, 577-8502 Japan; 5 Present Address: School of Pharmacy, Shujitsu University, 1-6-1 Nishigawara, Naka-ku, Okayama, 703-8516 Japan

**Keywords:** Bofutsushosan, Kampo medicine, Visceral fat, Ectopic fat, Obesity, Metabolic syndrome

## Abstract

This study aimed to compare fat accumulation in young and aged mice raised on a high-fat diet and to characterize the obesity-reducing effects of a Kampo medicine, bofutsushosan (BTS; fangfengtongshengsan in Chinese). Aged mice fed a high-fat diet containing 2% BTS extract for 28 days exhibited a significant reduction in weight gain and accumulation of visceral and subcutaneous fat, which were greater degree of reduction than those of the young mice. When the treatment period was extended to two months, the serum aspartate aminotransferase and alanine aminotransferase levels and the accumulation of fat droplets in the hepatocytes decreased. The mRNA expression of mitochondrial uncoupling protein 1 (UCP1) in the brown adipose tissue was significantly reduced in the aged mice compared to the young mice but increased by 2% in the BTS-treated aged mice. Additionally, the effect of BTS extract on oleic acid–albumin-induced triglyceride accumulation in hepatoblastoma-derived HepG2 cells was significantly inhibited in a concentration-dependent manner. Evaluation of the single crude drug extracts revealed that Forsythia Fruit, Schizonepeta Spike, and Rhubarb were the active components in BTS extract. These results suggest that BTS extract is effective against visceral, subcutaneous, and ectopic fats in the liver, which tend to accumulate with aging. Thus, BTS extract is useful in preventing and ameliorating the development of obesity and metabolic syndrome.

**Graphical Abstract:**

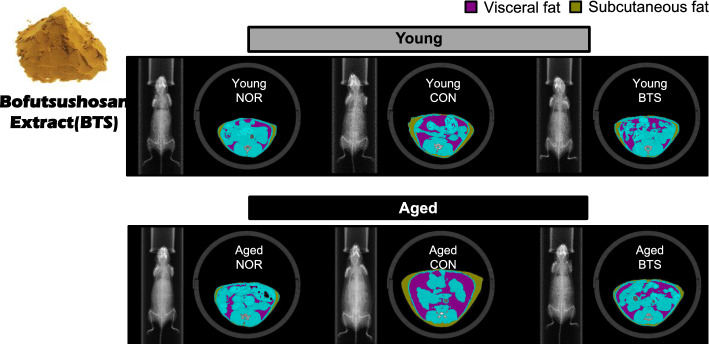

**Supplementary Information:**

The online version contains supplementary material available at 10.1007/s11418-024-01803-4.

## Introduction

Obesity due to fat accumulation is among the most common physical changes associated with aging after middle age [1, 2]. In particular, visceral obesity, wherein excessive fat accumulation occurs around the abdominal intestinal membranes, is a cause of lifestyle-related diseases, such as diabetes, dyslipidemia, and hypertension [3, 4]*.* If left untreated, these conditions can also lead to undesirable ectopic fat accumulation in non-adipose tissue [5]. Bofutsushosan (BTS; fangfengtongshengsan in Chinese) extract, a Kampo medicine listed in the Japanese Pharmacopoeia eighteenth edition (JP XVIII) [6], comprises 18 crude drugs: Japanese Angelica Root (*Angelicae Acutilobae Radix*, the root of *Angelica acutiloba* Kitagawa), Peony Root (*Paeoniae Radix*, the root of *Paeonia lactiflora* Pallas), Cnidium Rhizome (*Cnidii Rhizoma*, the rhizome of *Cnidium officinale* Makino), Gardenia Fruit (*Gardeniae Fructus*, the fruit of *Gardenia jasminoides* Ellis), Forsythia Fruit (*Forsythiae Fructus*, the fruit of *Forsythia suspensa* Vahl), Mentha Herb (*Menthae Herba*, the terrestrial part of *Mentha arvensis* Linné var. *piperascens* Malinvaud), Ginger (*Zingiberis Rhizoma*, the rhizome of *Zingiber officinale* Roscoe), Schizonepeta Spike (*Schizonepetae Spica*, the spike of *Schizonepeta tenuifolia* Briquet), Saposhnikovia Root and Rhizome (*Saposhnikoviae Radix*, the root and rhizome of *Saposhnikovia divaricata* Schischkin), Ephedra Herb (*Ephedrae Herba*, the terrestrial stem of *Ephedra sinica* Stapf), Rhubarb (*Rhei Rhizoma*, the rhizome of *Rheum palmatum* Linné), Sodium Sulfate (*Sal Mirabilis*, mineral substance composed of sodium sulfate), Atractylodes Rhizome (*Atractylodis Rhizoma*, the rhizome of *Atractylodes japonica* Koidzumi ex Kitamura), Platycodon Root (*Platycodi Radix*, the root of *Platycodon grandiflorus* A. De Candolle), Scutellaria Root (*Scutellariae Radix*, the root of *Scutellaria baicalensis* Georgi), Glycyrrhiza (*Glycyrrhizae Radix*, the root and stolon of *Glycyrrhiza uralensis* Fischer), Gypsum (*Gypsum Fibrosum*, natural hydrous calcium sulfate), and Aluminum Silicate Hydrate with Silicon Dioxide (*Kasseki*, mineral substance mainly composed of aluminum silicate hydrate and silicon dioxide). BTS has been indicated for the treatment of obesity in patients with excessive subcutaneous fat in the abdomen and constipation. In previous clinical reports, BTS has been shown to reduce visceral fat and improve insulin resistance in obese female patients with impaired glucose tolerance [7, 8], improve blood pressure variability in patients with essential hypertension associated with obesity [9], and reduce body mass index in participants with obesity [10]. In terms of the anti-obesity effects of BTS, different responses have been reported depending on the polymorphism of obesity-related genes [11]. Recently, Nakajima et al*.* conducted a clinical study on the abdominal visceral fat-reducing effects of the over-the-counter Kampo formulation of BTS [12]. Among non-clinical studies, the anti-obesity effects of BTS have been reported in several animal models for obesity or conditions, such as obesity with diabetes, hypertension, and non-alcoholic fatty liver [13–34]. The mechanisms of action of BTS have been reported to include activation of brown adipocytes and promotion of lipolysis in white adipocytes by β3 agonist action [8], promotion of lipid excretion into the stool [29], suppression of appetite via ghrelin [21], or a combination of these effects. Furthermore, the effect of BTS on the pre-disease state (*Mibyou* in Japanese) before the onset of metabolic syndrome has also been reported based on a dynamic network biomarker theory [35].

Previously, we reported the anti-obesity and anti-hyperlipidemic effects of natural compounds, such as acylated flavonol glycoside from rose hip (the fruit of *Rosa canina* L.) [36–38], sesquiterpene glycosides from artichoke (the leaves of *Cynara scolymus* L.) [39], saponins from tea flower (the flower buds of *Camellia sinensis* (L.) Kuntze) [40–42], daisy flower (the flowers of *Bellis perennis* L.) [43, 44], and the pericarps of *Sapindus rarak* DC. [45], oligostilbenes from the bark of *Shorea roxburghii* G. Don [46], and mate (the leaves of *Ilex paraguariensis* A. St.-Hil.) [47]. This study focused on age-related fat accumulation and its effects on visceral and ectopic fat. Additionally, to characterize the active crude drugs among the components of BTS, we performed in vitro tests to determine their effects on intracellular triglyceride (TG) levels in human hepatoblastoma-derived HepG2 cells.

## Materials and methods

### Preparation of BTS extract

The daily dosage of BTS for humans consists of 1.2 g of Japanese Angelica Root, 1.2 g of Peony Root, 1.2 g of Cnidium Rhizome, 1.2 g of Gardenia Fruit, 1.2 g of Forsythia Fruit, 1.2 g of Mentha Herb, 0.3 g of Ginger, 1.2 g of Schizonepetae Spica, 1.2 g of Schizonepeta Spike, 1.2 g of Saposhnikovia Root and Rhizome, 1.2 g of Ephedra Herb, 1.5 g of Rhubarb, 1.5 g of Sodium Sulfate, 2.0 g of Atractylodes Rhizome, 2.0 g of Platycodon Root, 2.0 g of Scutellaria Root, 2.0 g of Glycyrrhiza, 2.0 g of Gypsum, 3.0 g of Aluminum Silicate Hydrate with Silicon Dioxide. All the crude drugs met the grade standards of JP XVIII in accordance with the relevant regulations and were provided by Kobayashi Pharmaceutical Co., Ltd. (Osaka, Japan). The detailed information for each crude drug including the origin, producing area, and lot number is shown in Table S1.

**Fig. 1:**
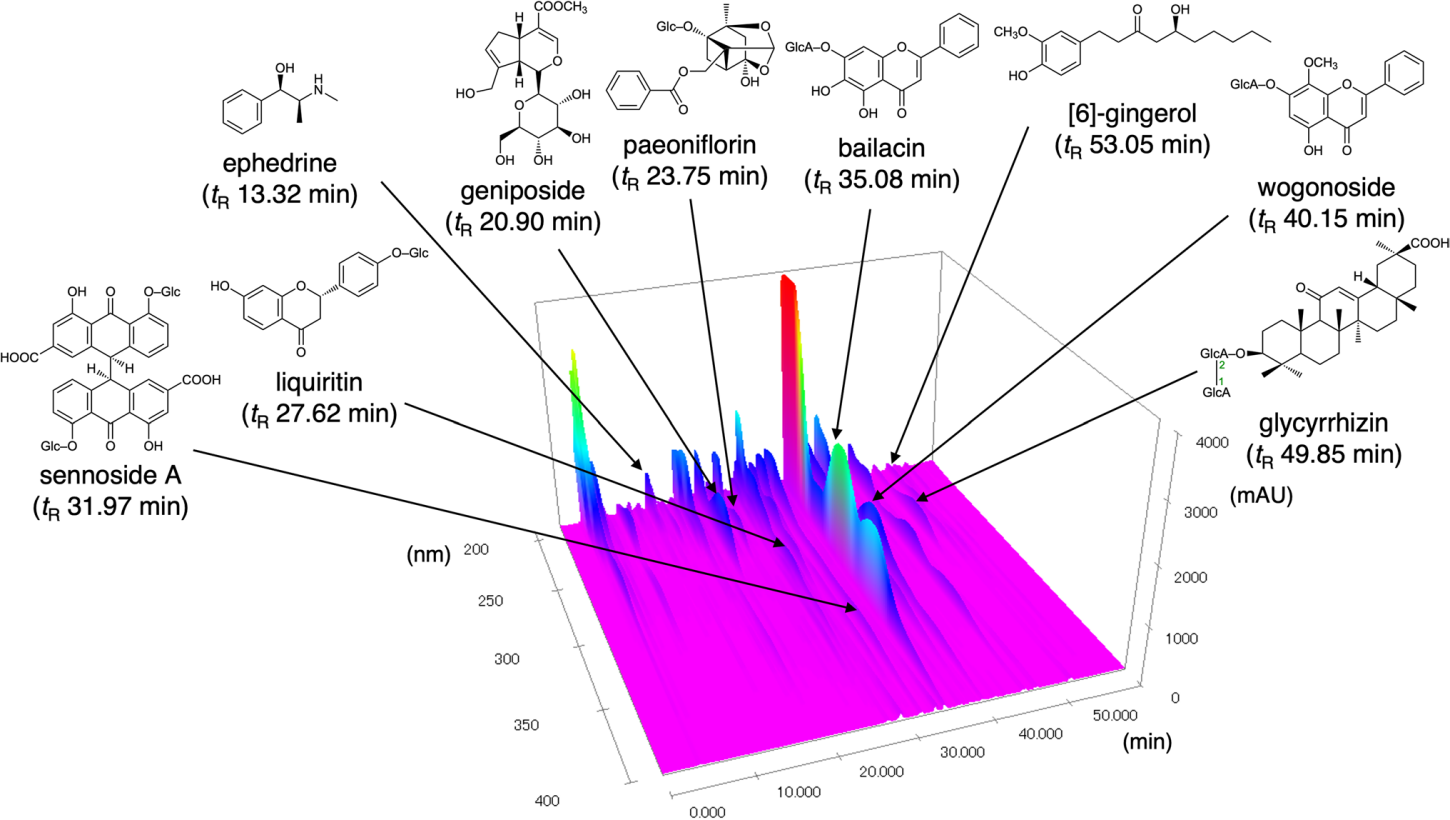
HPLC fingerprint of bofutsushosan (BTS) extract

For animal experiments, boiled water extract of BTS was produced as a bulk extract product (lot No. 1170418) at Kobayashi Pharmaceutical Co. Ltd. (Osaka, Japan). This extract met the standards of JP XVIII, and the contents of the marker compounds were as follows: 20.6 mg of paeoniflorin, 7.9 mg of total alkaloids (ephedrine and pseudoephedrine), 60.8 mg of baicalin, and 32.1 mg of glycyrrhizic acid in 5 g of BTS extract. The HPLC fingerprint of the BTS extract is shown in Fig. 1. Voucher specimens of single crude drugs and the extract were deposited at this company. For cell experiments, the extracts of BTS and each single crude drug were prepared in our laboratory.

### HPLC fingerprint of BTS extract

BTS extract (500 mg) was suspended in 50% aqueous methanol (10 mL) and sonicated for 30 min. After centrifugation (3000 rpm, 10 min), the supernatant was filtered through a syringe filter (0.45 μm) to obtain the sample solution. The sample solution (10 μL) was injected into HPLC with the following conditions [column: TSK-Gel ODS-100 V (4.6 × 250 mm, i.d., 5 μm); mobile phase: 10 mM aqueous phosphoric acid–CH_3_CN 95:5 (0 min)–40:60 (60 min), linear gradient; flow rate: 0.8 mL/min; column temperature: 40 °C; detection: 200–400 nm by a photodiode array detector]. Some peaks were identified as ephedrine (*t*_R_ 13.32 min from Ephedra Herb), geniposide (*t*_R_ 20.90 min from Gardenia Fruit), paeoniflorin (*t*_R_ 23.75 min from Peony Root), liquiritin (*t*_R_ 27.62 min from Glycyrrhiza), sennoside A (*t*_R_ 31.97 min from Rhubarb), bailacin (*t*_R_ 35.08 min from Scutellaria Root), wogonoside (*t*_R_ 40.15 min from Scutellaria Root), glycyrrhizin (*t*_R_ 49.85 min from Glycyrrhiza), and [6]-gingerol (*t*_R_ 53.05 min from Ginger) by comparing the retention times and UV spectra with those of standard samples.

### Preparation of single crude drug extracts

For preparation of the water and methanol extracts of each single crude drug, 1 g of the powdered crude drug was extracted with distilled water or methanol (15 mL) under boiling water for 60 min or sonicated for 30 min. After centrifugation (3000 rpm, 10 min), the supernatant was dried under reduced pressure to obtain the extract. The extraction yields of the water and methanol extracts of each crude drug are shown in Table S1 (the HPLC fingerprints of the methanol extracts of each single crude drug, see Figs. S1–15). The stock solution of each extract for in vitro assay was prepared as a DMSO solution at a concentration of 0.1 g/mL and stored at − 20 °C until use. Voucher specimens of the crude drugs were deposited at this company and the Pharmaceutical Research and Technology Institute, Kindai University.

### Animals and treatments

Male C57BL/6 *J* mice were purchased from CLEA Japan, Inc. (Tokyo, Japan). High-fat (HFD32) and standard (CE-2) diets were purchased from CLEA Japan. HFD32 contained 24.5% milk casein, 5.0% egg white, 0.43% L-cystine, 15.88% beef tallow, 20% safflower oil, 5.5% crystalline cellulose, 8.25% maltodextrin, 6.928% lactose, 6.750% sucrose, 1.4% AIN93 vitamin mix, 5.0% AIN93G mineral mix, 0.36% choline bitartrate, and 0.002% tertiary-butyl hydroquinone. The total lipid and the cholesterol contents in the HFD32 were 32% and 0.019%, respectively. The animals were housed at a constant temperature of 23 ± 2 °C, 55 ± 15% humidity, and 12 h of illumination per day. The animal studies were conducted in accordance with the institutional guidelines for animal experiments at Kobayashi Pharmaceutical Co. Ltd. and all experimental protocols were approved by the institutional committee at Kobayashi Pharmaceutical Co. Ltd.

### Effects of 4-week BTS extract administration on weight gain, visceral fat, liver weight, and plasma biochemicals in mice fed a high-fat diet

Eight-week-old (Young group) or 42–62-week-old male mice (Aged group) were pre-reared with a standard CE-2 diet for 1 week and then separated into three groups (day 0): normal (NOR; reared with CE-2 diet), control (CON; HFD32 diet), and BTS extract-treated (BTS; 2% BTS extract-containing HFD32 diet) groups. Each mouse was reared individually with free access to food and water for 28 days. Food and water intake, and individual body weights were monitored throughout the experimental period. On day 24, visceral and subcutaneous fat amounts were measured using an experimental X-ray computed tomography (CT) instrument (Latheta LCT-100, Hitachi Healthcare, Tokyo, Japan) under isoflurane-induced anesthesia by scanning from the ensiform cartilage to the sacral bone at 1.5-mm intervals. On day 28, mice were anesthetized by isoflurane inhalation, the abdomen was opened, blood was collected from the abdominal vena cava, and the weights of tissues were measured. The serum concentrations of glucose, TG, non-esterified fatty acid, total cholesterol, aspartate aminotransferase (AST), alanine aminotransferase (ALT), and alkaline phosphatase were measured using a 7180 Clinical Analyzer (Hitachi High-Tech Corporation, Tokyo, Japan).

### Effects of two-month BTS extract administration on histopathology of liver tissue in mice fed a high-fat diet

Eight-week-old (Young group) or 63–64-week-old male mice (Aged group) were pre-reared with standard CE-2 diet for 1 week and then divided into two groups (day 0): control (CON; reared with HFD32 diet), and BTS extract-treated (BTS; 2% BTS extract-containing HFD32 diet) groups. Each mouse was reared individually with free access to food and water for 62 days, and their intake and body weights were monitored throughout the experimental period. On day 62, mice were anesthetized via isoflurane inhalation, the abdomen was opened, blood was collected from the abdominal vena cava, and the weights of tissues were measured. After removing the liver, *ca.* 50 mg of the liver tissue was homogenized with 5% nonidet-P-40 (500 µL; Nacalai Tesque Inc., Kyoto, Japan), and the TG and protein contents in the homogenate were determined using a Triglyceride E-test Wako kit (FUJIFILM Wako Pure Chemical Co.) and a BCA protein Assay Kit (Thermo Fisher Scientific Inc., Waltham, MA, USA), respectively.

The liver tissue was fixed with formaldehyde and embedded in a paraffin block, sectioned, and stained with hematoxylin and eosin for histopathological analysis. The serum biochemicals were measured using the 7180 Clinical Analyzer. The total RNA was isolated using RNAiso Plus (Takara Bio Inc., Ohtsu, Japan). The first-strand cDNA was synthesized from 250 ng of total RNA using ReverTra Ace qPCR Master Mix with DNA remover (Toyobo, Japan) according to the manufacturer’s instructions. Quantitative PCR was performed in a Takara-bio TP800 Thermal Cycler Dice real-time system (Takara Bio Inc.) with SYBR Green PCR Master Mix. Relative mRNA expression was assessed by the ΔΔCt method using *β*-actin as an endogenous control to normalize samples. The following primer sequences were used in the present study: *Ucp1*, forward primer 5′-TCA GGG CTG AGT CCT TTT GT-3′ and reverse primer 5′-CTG AAA CTC CGG CTG AGA AG-3′; *β*-actin, forward primer 5′-CAT CCG TAA AGA CCT CTA TGC CAA C-3′ and reverse primer 5′-ATG GAG CCA CCG ATC CAC A-3′.

### Cell culture

The HepG2 cells (RCB1648, Riken Cell Bank, Tsukuba, Japan) were maintained in Minimum Essential Medium Eagle (MEM; Sigma-Aldrich Co. LLC., St. Louis, MO, USA) containing 10% fetal bovine serum, 1% MEM non-essential amino acids (FUJIFILM Wako Pure Chemical Co.), penicillin G (100 units/mL), and streptomycin (100 µg/mL) at 37 °C and 5% CO_2_ in the atmosphere.

### Effects on oleic acid–albumin-induced TG accumulation in HepG2 cells

Effects on lipid accumulation inhibitory activity in oleic acid–albumin-induced HepG2 cells were evaluated as previously described [48–50]. The cells were inoculated in a 48-well tissue culture plate (10^5^ cells/well in 200 µL/well in MEM). After 20 h, the medium was replaced with 200 µL/well of Dulbecco’s Modified Eagle’s Medium (DMEM) containing 1000 mg/L glucose, a test sample with or without 5% (v/v) oleic acid–albumin (Sigma-Aldrich Co. LLC., St. Louis, MO, USA, final concentration: 0.158 mmol oleic acid and 5.25 mg/mL BSA). The cells were cultured for 4 days, replacing the medium with a fresh one every 2 days. Subsequently, the medium was removed, and the cells were homogenized in distilled water (105 µL/well) via sonication. The TG and protein contents in the homogenate were determined using commercial kits (vide supra). Data were expressed as the percentage of control of TG/protein (µg/mg). Each test compound was dissolved in DMSO and the solution was added to the medium (the final DMSO concentration was 0.5%). A nuclear receptor peroxisome proliferator-activated receptor α agonist, bezafibrate (Tokyo Chemical Industry Co., Ltd., Tokyo, Japan), was used as a reference compound.

### Effects on TG contents in high-glucose-pretreated HepG2 cells

Effects on TG metabolism-promoting activity in high-glucose-pretreated HepG2 cells were evaluated as previously described [48, 51–53]. HepG2 cells were inoculated in a 48-well tissue culture plate (10^5^ cells/well in 150 µL/well in MEM). After 20 h, the medium was replaced with 150 µL/well of high-glucose-containing DMEM and cultured for 6 days, with the medium being replaced every 2 days. After lipid accumulation, the medium was transferred to low-glucose-containing DMEM (150 µL/well) and a test sample, and the cells were cultured. After 20 h, the TG and protein contents in the cells were determined in the same manner described above. Data were expressed as the percentage of control of TG/protein (µg/mg). Each test compound was dissolved in DMSO and added to the medium (the final DMSO concentration was 0.5%). An anti-diabetic agent, metformin (FUJIFILM Wako Pure Chemical Co.), was used as a reference compound.

### Statistical analysis

Values are expressed as mean ± standard error. A one-way analysis of variance followed by a Dunnett’s test was used for statistical analysis. Probability (*p*) values of less than 0.05 were considered significant.

## Results

### Animal experiments

**Fig. 2:**
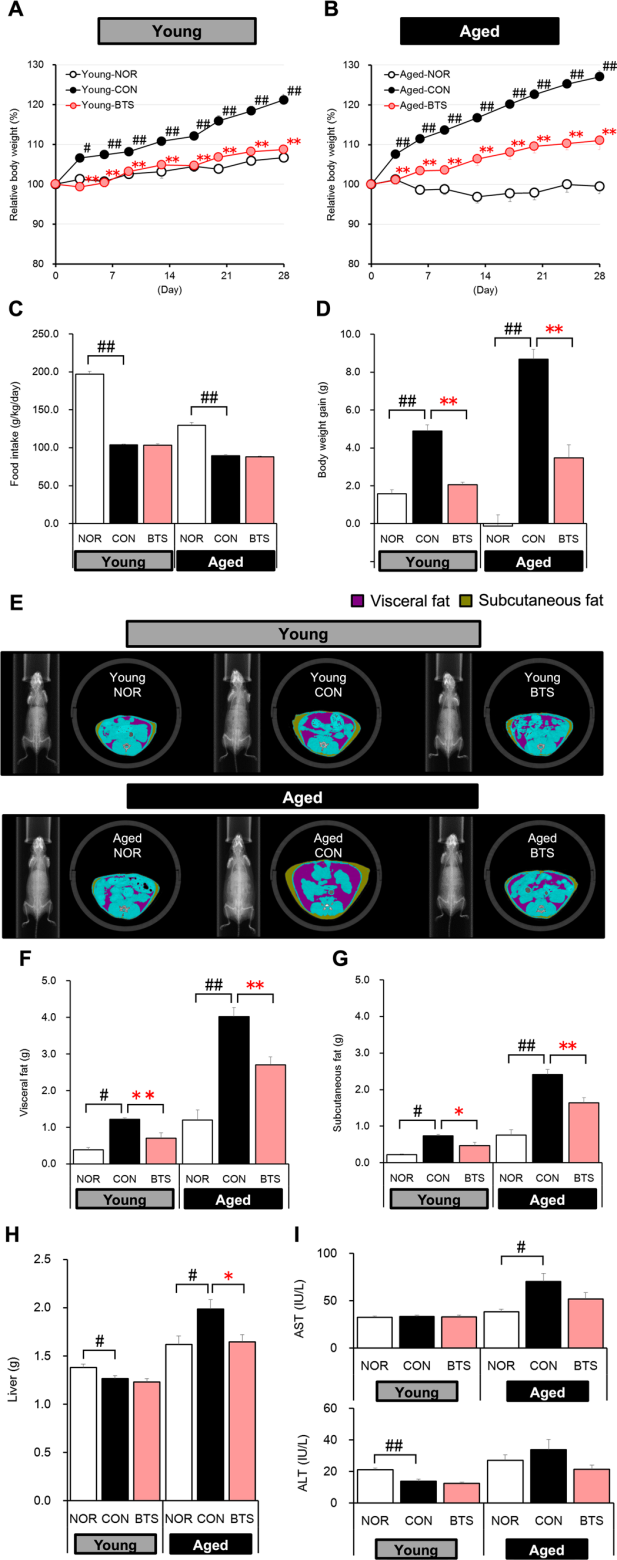
Effects of 4 weeks of bofutsushosan (BTS) extract administration on the physical and biochemical parameters of mice fed a high-fat diet Relative body weights of Young (**A**) and Aged groups (**B**), food intake (**C**), body weight gain (**D**), representative image of X-ray computed tomography (CT) of mice (**E**), and amount of visceral (**F**) and subcutaneous fat (**G**) analyzed from images obtained on day 24, and liver weights (**H**) and function tests (**I**, serum ALT and AST) on day 28. NOR: normal group (CE-2 diet); CON: control group (HFD32 diet); BTS: 2% BTS extract-containing HFD32 diet. Mean ± S.E. (*n* = 5–8). ^#^*p* < 0.05, ^##^*p* < 0.01 vs. Normal, *p < 0.05, **p < 0.01 vs. Control (*t*-test)

The physical and biochemical parameters of 4-week administration of BTS extract to young and aged mice fed a high-fat diet are shown in Table S2. As shown in Fig. 2A and B, the body weights of young (Young-CON) and aged control (Aged-CON) groups fed the HFD32 diet for 28 days were found to have increased to approximately 121% [Young-CON: day 0 (23.3 ± 0.4 g); day 28 (28.2 ± 0.4 g)] and 127% [Aged-CON: day 0 (32.1 ± 0.7 g); day 28 (40.8 ± 1.1 g)], respectively. However, body weight gain was reduced in both young and aged BTS-treated groups {Young-BTS: 109% [day 0 (23.7 ± 0.5 g); day 28 (25.8 ± 0.4 g)]; Aged-BTS: 111% [day 0 (31.7 ± 0.7 g); day 28 (35.2 ± 1.0 g)]}. No significant difference in the average food intake was observed in the control and BTS-treated groups during the experimental period [Young-CON (2.7 ± 0.0 g/day) vs. Young-BTS (2.6 ± 0.0 g/day); Aged-CON (3.3 ± 0.1 g/day) vs. Aged-BTS (3.0 ± 0.1 g/day) (Fig. 2C)]. Additionally, average water intake values were similar among these groups (data not shown). The result is summarized in Fig. 2D. The body weight gains for the BTS-treated groups [body weight gain for Young-BTS (2.1 ± 0.1 g); Aged-BTS (3.5 ± 0.7 g)] were found to be significantly reduced compared to those of the control groups for both the young and aged groups [Young-CON (4.9 ± 0.3 g); Aged-CON (8.7 ± 0.5 g)].

The amounts of visceral, subcutaneous, and total fat were analyzed using representative X-ray CT photos of mice taken on day 24 (Fig. 2E). The image analysis showed that the amounts of visceral [Aged-NOR (1.20 ± 0.28 g); Aged-CON (4.02 ± 0.25 g); Young-NOR (0.39 ± 0.05 g): Young-CON (1.22 ± 0. 44 g) (Fig. 2F)] and subcutaneous [Aged-NOR (0.75 ± 0.15 g); Aged-CON (2.42 ± 0.13 g); Young-NOR (0.22 ± 0.02 g); Young-CON (0.73 ± 0.05 g) (Fig. 2G)] fats were significantly higher in the aged than in the young groups. The amounts of visceral and subcutaneous fats in the young and aged BTS-treated groups were significantly lower than those in the control groups [visceral fat: Aged-BTS (2.71 ± 0.21 g); Young-BTS (0.70 ± 0.15 g); subcutaneous fat: Aged-BTS (1.63 ± 0.14 g); Young-BTS (0.47 ± 0.09 g)].

On day 28, mice were sacrificed, and liver tissue and blood were collected. For the aged group, the weights of the liver tissue in the BTS-treated group were significantly lower than those in the control group [Aged-CON (1.98 ± 0.10 g); Aged-BTS (1.64 ± 0.08 g) (Fig. 2H)]. Additionally, the BTS-treated group showed reduced levels of the serum hepatic biomarkers AST and ALT compared to the corresponding control group [AST: Aged-CON (70.1 ± 8.6 IU/L); Aged-BTS (52.0 ± 6.5 IU/L); ALT: Aged-CON (33.9 ± 6.4 IU/L); Aged-BTS (21.3 ± 2.7 IU/L) (Fig. 2I)].

**Fig. 3:**
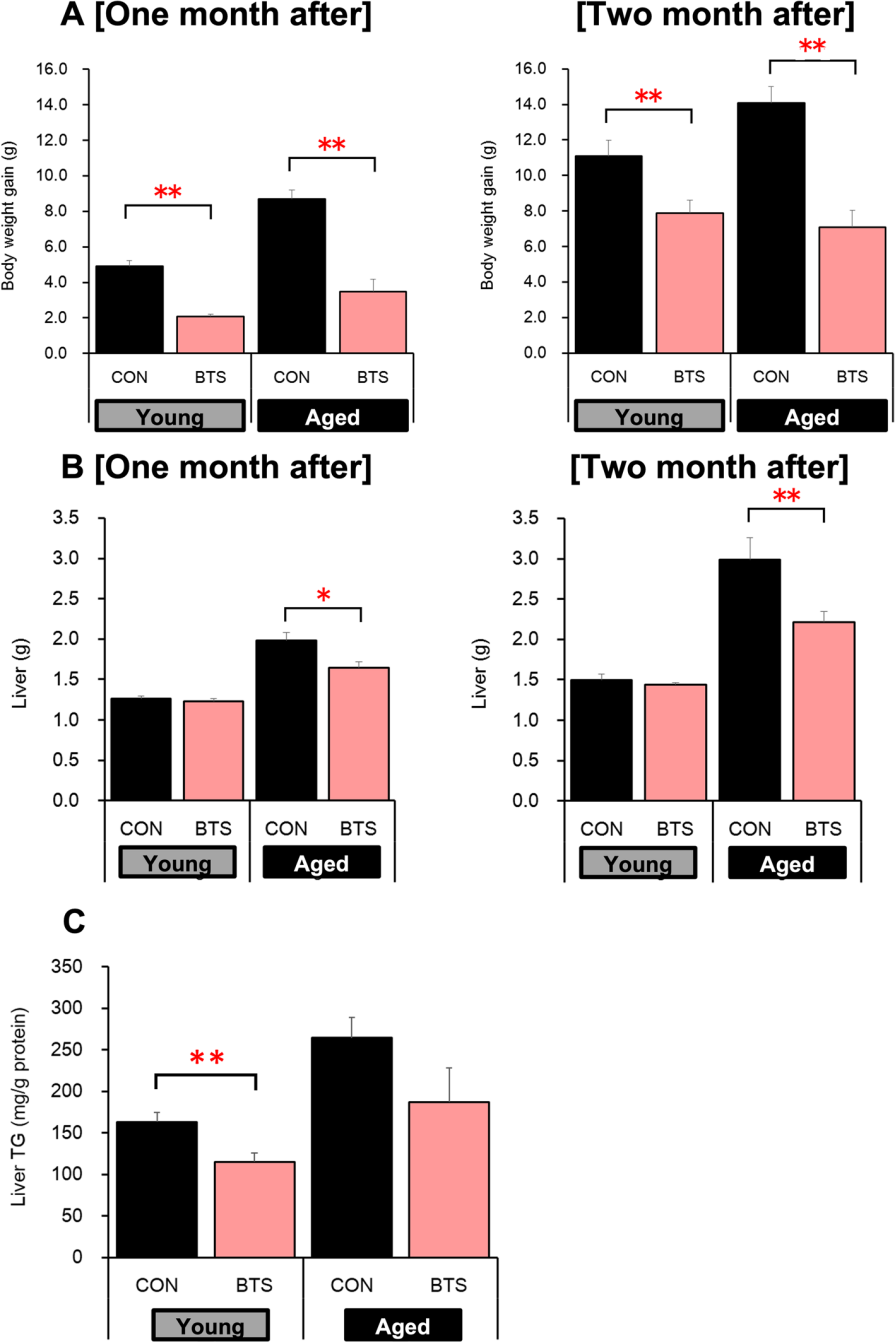

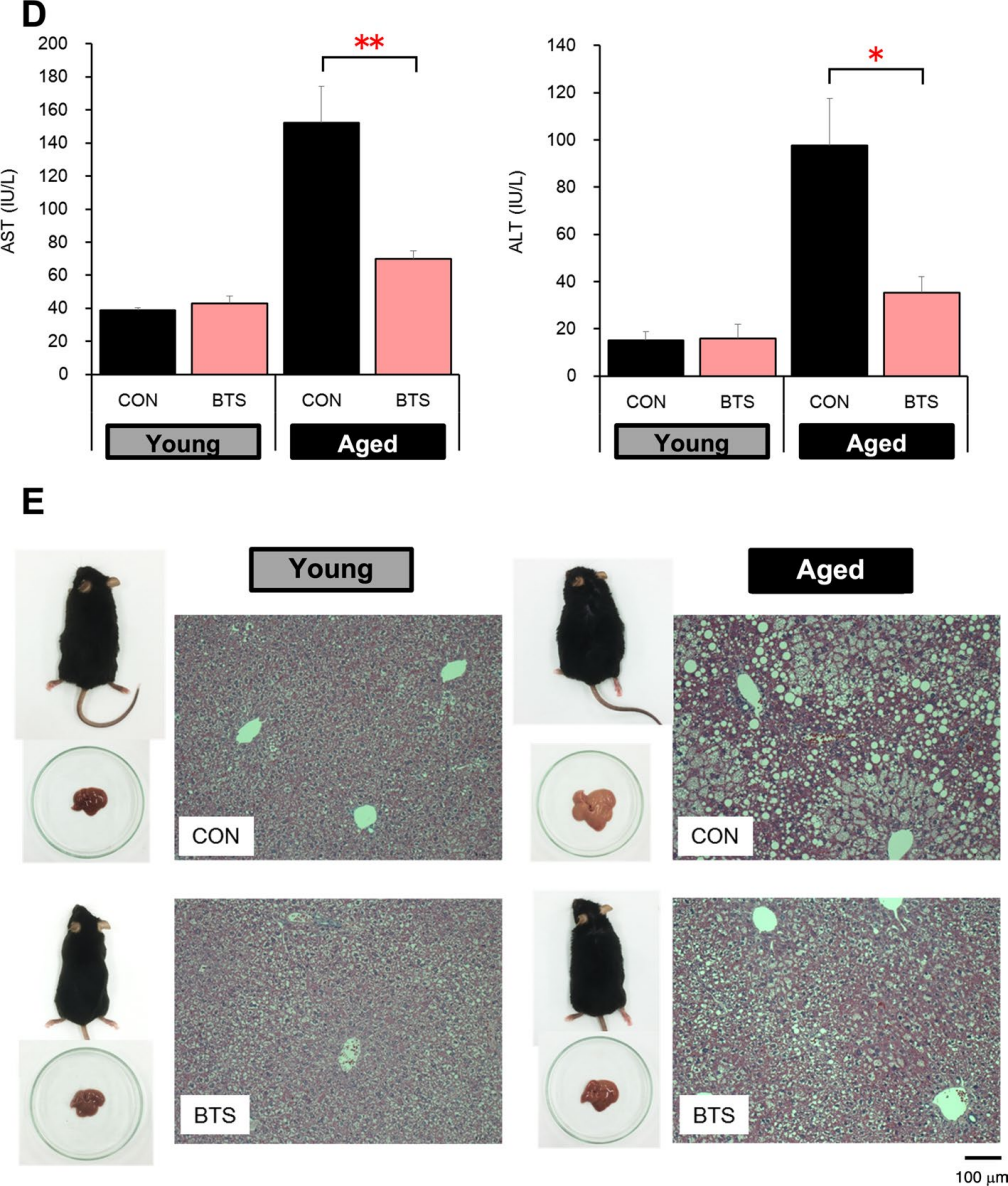
Effects of two months of bofutsushosan (BTS) extract administration on histopathological images of liver tissue in mice fed a high-fat diet body weight gain (**A**), liver weight (**B**) and TG (**C**), serum ALT and AST levels (**D**), and histopathological liver image in hematoxylin and eosin staining (**E**) at day 62. CON: control group (HFD32 diet); BTS: 2% BTS extract-containing HFD32 diet. Mean ± S.E. (*n* = 5–8). ^#^*p* < 0.05, ^##^*p* < 0.01 vs. Normal, *p < 0.05, **p < 0.01 vs. Control (*t*-test)

**Fig. 4:**
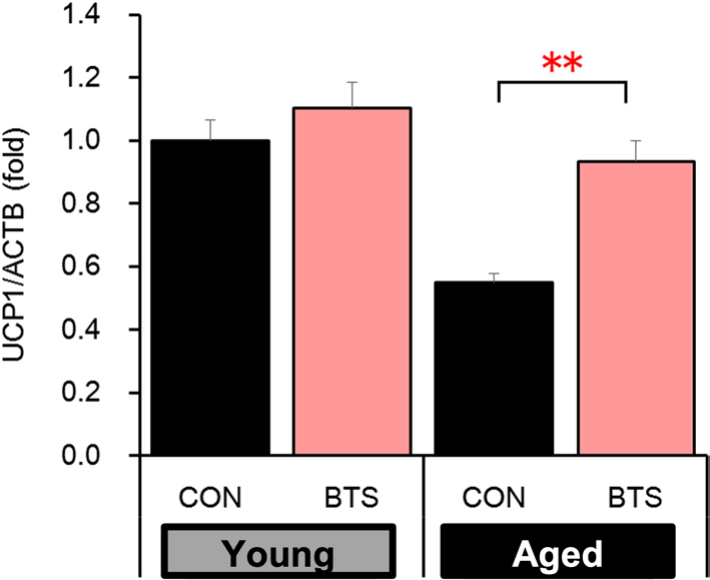
Effects of two months of bofutsushosan (BTS) extract administration on mitochondrial uncoupling protein 1 mRNA level in brown adipose tissue of mice fed a high-fat dietCON: control group (HFD32 diet); BTS: 2% bofutsushosan (BTS) extract-containing HFD32 diet. Mean ± S.E. (*n* = 5–8). **p < 0.01 vs. Control (*t*-test)

Subsequently, the aforementioned in vivo study was extended to two months (62 days) to evaluate the reproducibility and scrutinize the effects on fat accumulation in the liver (see Table S3). As shown in Fig. 3A, the BTS extract significantly reduced body weight gain in both young and aged mice [weight gain over 62 days: Young-CON (11.1 ± 0.9 g); Aged-CON (14.1 ± 0.9 g); Young-BTS (7.9 ± 0.7 g); Aged-BTS (7.1 ± 1.0 g)] without significantly affecting in their food and water intakes. The liver weights of the aged BTS-treated group were significantly reduced compared to the corresponding control group [Aged-CON: 2.99 ± 0.28 g; Aged-BTS: 2.21 ± 0.13 g (Fig. 3B)]. Furthermore, the TG content in the liver was reduced (Aged-CON: 264.2 ± 24.6 mg/g protein; Aged-BTS: 187.1 ± 40.9 mg/g protein), as shown in Fig. 3C. Serum AST and ALT levels in both aged BTS-treated groups [AST (70.0 ± 4.9 IU/L); ALT (35.3 ± 4.3 IU/L)] were significantly reduced compared to those of the aged control group [AST (152.4 ± 22.0 IU/L); ALT (97.8 ± 23.1 IU/L)], as shown in Fig. 3D. Hematoxylin and eosin-stained liver sections were prepared to further verify the histopathological analysis. As shown in Fig. 3E, the aged control group showed accumulation of numerous fat droplets between tissues, whereas the aged BTS-treated group showed reduced accumulation. The mRNA expression of mitochondrial UCP1 in the brown adipose tissue (BAT) was significantly decreased in the aged mice compared to the young mice but increased by 2% in the aged BTS-treated mice (Fig. 4).

### In vitro lipid accumulation inhibitory and metabolism-promoting activities of single crude drug extracts in HepG2 cells

**Fig. 5:**
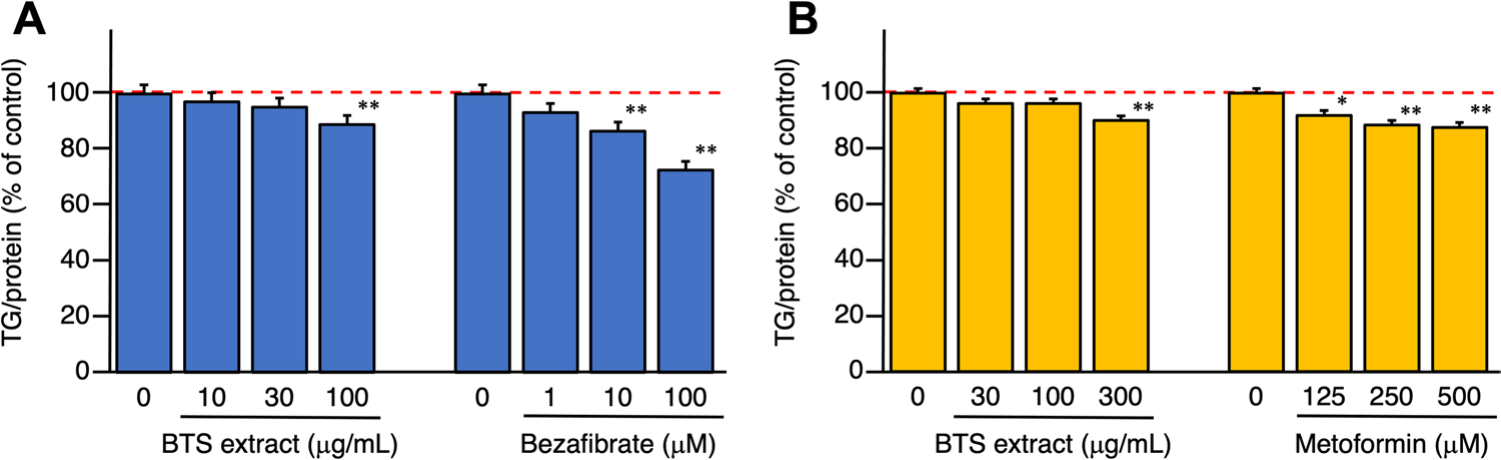
Effects of bofutsushosan (BTS) extract on intracellular triglyceride contents in oleic acid–albumin-induced and high glucose-pretreated HepG2 cells. Effects on oleic acid–albumin-induced triglyceride accumulation in HepG2 cells (**A**) and triglyceride contents in high glucose-pretreated HepG2 cells (**B**). Each value represents the mean ± S.E. (*n* = 4).Significantly different from the control, *p < 0.05, **p < 0.01

Intracellular TG contents in hepatoblastoma-derived HepG2 cells were significantly reduced by adding BTS extract in a dose-dependent manner to the medium with oleic acid–albumin-induced (Fig. 5A) and to high glucose-pretreated culture conditions (Fig. 5B). As a marker of cytotoxicity in HepG2 cells, a protein assay was performed. This assay is one of the common cytotoxicity tests in toxicology research [54, 55] and a good correlation between the cell number and the protein content has been reported [56]. The BTS extract did not exhibit cytotoxicity in the concentration range studied (see Tables S4 and S5).

The water and methanol extracts of each crude drug were prepared to identify the active crude drug component in BTS. The extracts of the prepared crude drugs are described in Table S1. The quantities of the water extracts of Gypsum (*Gypsum Fibrosum*, natural hydrous calcium sulfate) and Aluminum Silicate Hydrate with Silicon Dioxide (*Kasseki*, mineral substance, mainly composed of aluminum silicate hydrate and silicon dioxide) and the methanol extracts of Sodium Sulfate (*Sal Mirabilis*), Gypsum, and Aluminum Silicate Hydrate with Silicon Dioxide were too low. We evaluated the effects of 16 water and 15 methanol extracts on TG accumulation inhibitory and TG metabolism-promoting in HepG2 cells.

**Fig. 6:**
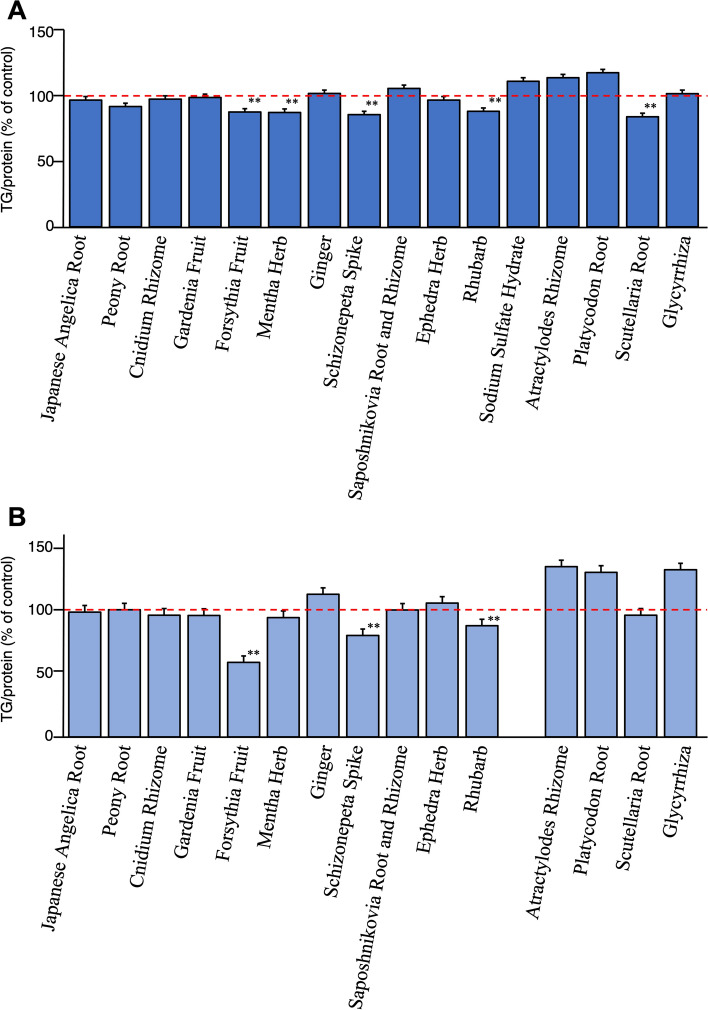
Effects of crude drug extracts of each component of bofutsushosan (BTS) extract on oleic acid–albumin-induced triglyceride accumulation in HepG2 cellsH_2_O Extract (100 µg/mL) (**A**) and methanol extract (30 µg/mL) (**B**).Each value represents the mean ± S.E. (*n* = 4). Significantly different from the control, *p < 0.05, **p < 0.01

Among the single crude drug extracts, the water extracts of Forsythia fruit (% of control for TG/protein at 100 µg/mL: 88.9 ± 4.4), Mentha Herb (88.6 ± 1.4), Schizonepeta Spike (86.9 ± 3.0), Rhubarb (89.5 ± 1.6), and Scutellaria Root (85.3 ± 3.8) were found to significantly inhibit TG accumulation (Fig. 6A), with no detectable cytotoxic effects (data not shown). The methanol extracts of Forsythia Fruit (% of control for TG/protein at 30 µg/mL: 59.0 ± 3.0), Schizonepeta Spike (80.4 ± 1.2), and Rhubarb (88.2 ± 4.3) were also found to significantly inhibit TG accumulation, as shown in Fig. 6B (see Table S4).

**Fig. 7:**
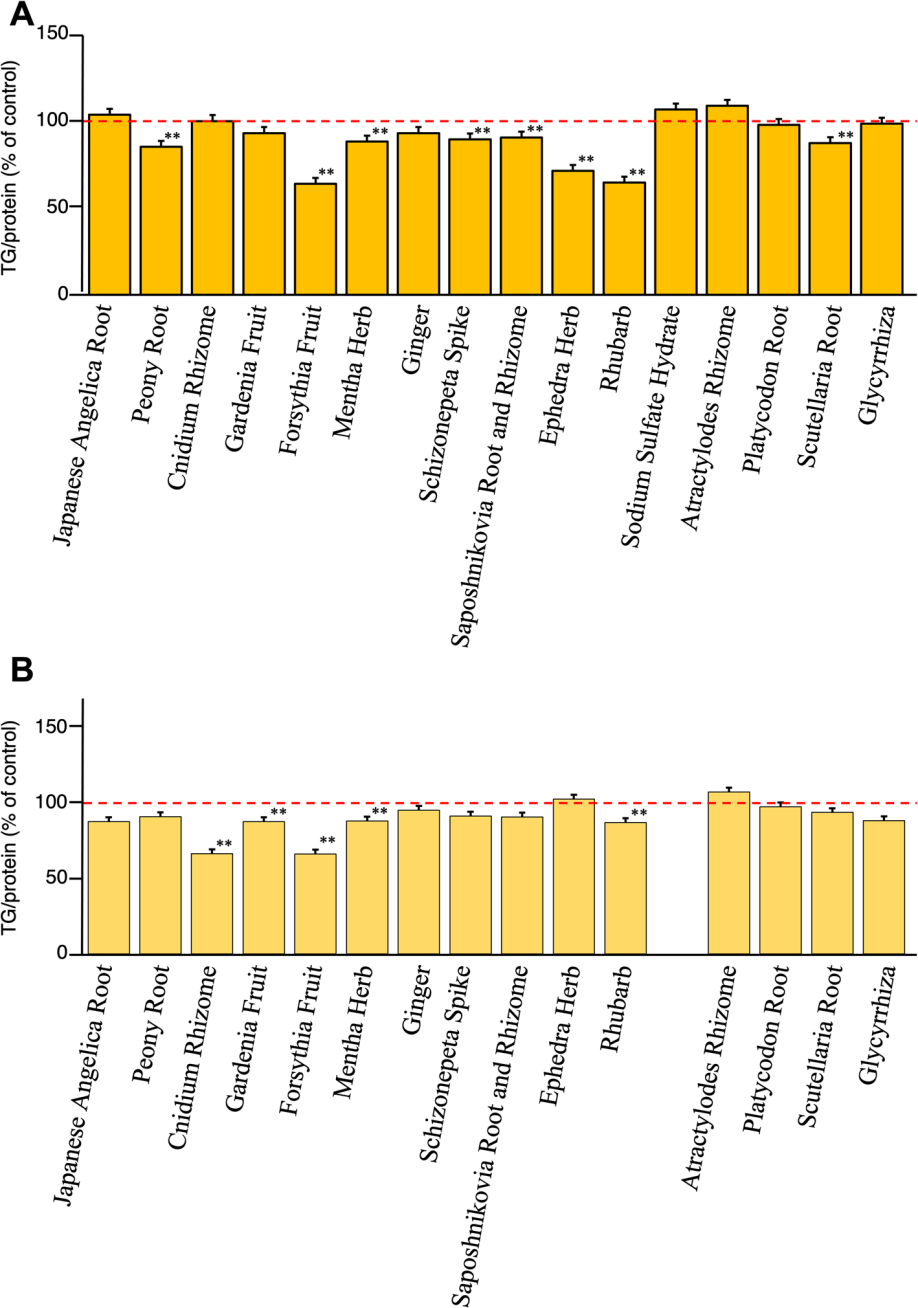
Effects of crude drug extracts of each component of bofutsushosan (BTS) on triglyceride contents in high glucose-pretreated HepG2 cells H_2_O Extract (300 µg/mL) (**A**) and methanol extract (100 µg/mL) (**B**) Each value represents the mean ± S.E. (*n* = 4) Significantly different from the control, *p < 0.05, *p < 0.01

Figure 7A and B shows the effects of the single crude drug extracts on TG metabolism promotion determined by monitoring the intracellular TG contents in high-glucose-pretreated HepG2 cells. The results indicate that the water extracts of Forsythia Fruit (% of control for TG/protein at 300 µg/mL: 63.8 ± 0.8), Ephedra Herb (71.3 ± 1.8), and Rhubarb (64.6 ± 1.4) and the methanol extracts of Cnidium Rhizome (% of control for TG/protein at 30 µg/mL: 66.4 ± 5.1) [57], Forsythia Fruit (66.2 ± 2.0), and Scutellaria Root (at 100 µg/mL: 77.6 ± 2.0), Rhubarb (79.1 ± 2.9), and Glycyrrhiza (52.5 ± 1.0) significantly promoted TG metabolism without cytotoxic activity under the effective concentrations (see Table S5).

## Discussion

The present study demonstrated that BTS extract reduced body weight gain in mice fed a high-fat diet. After being fed a high-fat diet for 28 days, the weight gains of the aged mice (43–63 weeks old) were greater than those of the young mice (6 weeks old), suggesting that fat accumulation becomes easier with age. Both weight gain and accumulation of visceral and subcutaneous fat in the 2% BTS extract-treated group of the aged mice were significantly reduced compared to that in the control group of the aged mice. Additionally, liver weights increased in aged mice compared to young mice, suggesting ectopic fat accumulation. Furthermore, AST and ALT levels were significantly increased in the aged mice (64–85 weeks old) when the treatment period was extended to two months. Histological analysis showed that many fat droplets were deposited in the hepatocytes. Furthermore, fat accumulation in the liver was reduced and the serum AST and ALT levels decreased in the 2% BTS extract-treated group of the aged mice.

As previous related studies, Kim et al*.* reported that the susceptibility of hepatic inflammation and liver fibrosis were found to significantly increase caused by aging in response to high-fat diet (HFD) in mice [58]. Similarly, Fontana et al. reported that the development of HFD-induced murine steatohepatitis promoted by aging [59]. Therefore, aging with HFD intake considered to be a risk factor that promotes inflammation and fibrosis of liver. Indeed, in our study, we observed increased the liver damage in aged mice compared to young mice, whereas the aged BTS extract-treated mice effectively suppressed the liver damage.

Soro-Arnaiz et al*.* identified that aged adipocytes have decreased energy expenditure by intracellular mitochondria and promoted lipid accumulation than that of young adipocytes [60]. BAT is the major thermogenic organ used for adaptive thermogenesis in response to environmental challenges, such as diet and cold. Wang et al. reported that the decrease amount of BAT might be associated with accumulation of visceral fat [61]. Especially in old people, the amount of BAT inversely correlated with body mass index [62]. BAT is highly vascularized and richly innervated with the sympathetic nervous system, and brown adipocytes contain a high density of mitochondria which express GDP binding protein, UCP1, on their inner membranes [8]. The thermogenic ability of BAT is conferred by UCP1, a BAT-specific transport protein of the inner mitochondrial membrane [63, 64]. Therefore, in this study, we investigated the effect of BTS extract on the expression of UCP1 in BAT, which decreases with age. As a result, the expression of UCP1 decreased in aged mice compared to young mice, and administration of BTS extract significantly suppressed this decrease in aged mice. Consequently, it was thought that BTS extract exerted an anti-obesity effect by increasing fat metabolism, which has decreased with age, and also effectively suppressed the accumulation of ectopic fat in the liver.

The reducing effect of the BTS extract on liver fat accumulation was reproduced by in vitro assays, such as the effects on oleic acid–albumin-induced TG accumulation and TG contents in high glucose-pretreated HepG2 cells. By adding oleic acid–albumin to the medium cultured of HepG2 cells, accumulation of the intracellular TG content measured using a commercial kit was found to observe by comparison with that of the condition of the additive-free (see Table S4) [47–50, 65, 66]. It was also marked by lipid droplet accumulation as measured by Oil red O staining-based colorimetric assay [67–69]. On the other hand, regarding the high glucose-pretreated model in HepG2, we have established the culture condition monitored by the intracellular TG content (see Figure S16). The effect of BTS extract on oleic acid–albumin-induced TG accumulation in hepatoblastoma-derived HepG2 cells was significantly inhibited at a concentration of 100 µg/mL. Among the crude drug components of BTS, Forsythia Fruit, Schizonepeta Spike, and Rhubarb were found to be active in both water (100 µg/mL) and methanol extracts (30 µg/mL). Additionally, in the high-glucose-pretreated HepG2 cells incubated with BTS extract, the TG levels in cells were significantly reduced at a concentration of 100 µg/mL. Among them, the methanol extracts of Cnidium Rhizome [57] and Forsythia Fruit were found to show potent TG metabolism-promoting activity. These findings indicate that the combination of multiple crude drugs prescribed for BTS is important for its anti-obesity effects.

The metabolic dysregulation associated with obesity is similar to that observed in aging [70, 71]. The metabolic risk related to obesity is, at least in part, associated with common diabetes and cardiovascular disease risk factors, including hyperglycemia, hypertension, and dyslipidemia [71, 72]. Therefore, by the above-mentioned evidence, it was suggested that BTS extract can effectively inhibit the development of visceral, subcutaneous, and ectopic fat in the liver, which tend to accumulate with aging, and helps prevent obesity and metabolic syndrome. To elucidate the detailed mechanisms of actions of BTS extract, further research on the active constituents of individual crude drug components is needed and will be discussed in a future study.

## Supplementary Information

Below is the link to the electronic supplementary material.

Supplementary file1 (PDF 9883 KB)
