# Correction: Ameliorative effect of bofutsushosan (Fangfengtongshengsan) extract on the progression of aging-induced obesity

**DOI:** 10.1007/s11418-025-01899-2

**Published:** 2025-03-25

**Authors:** Takafumi Saeki, Saya Yamamoto, Junji Akaki, Takahiro Tanaka, Misaki Nakasone, Hidemasa Ikeda, Wei Wang, Makoto Inoue, Yoshiaki Manse, Kiyofumi Ninomiya, Toshio Morikawa

**Affiliations:** 1 Central R&D Laboratory, Kobayashi Pharmaceutical Co., Ltd, 1-30-3 Toyokawa, Ibaraki Osaka, 567-0057 Japan; 2 Pharmaceutical Research and Technology Institute, Kindai University, 3-4-1 Kowakae, Higashi-osaka, Osaka, 577-8502 Japan; 3 Laboratory of Medicinal Resources, School of Pharmacy, Aichi Gakuin University, 1-100 Kusumoto-cho, Chikusa-ku Nagoya, 464-8650 Japan; 4 Antiaging Center, Kindai University, 3-4-1 Kowakae, Higashi-osaka, Osaka, 577-8502 Japan; 5 Present Address: School of Pharmacy, Shujitsu University, 1-6-1 Nishigawara, Naka-ku, Okayama, 703-8516 Japan

**Correction: Journal of Natural Medicines (2024) 78:576–589** 10.1007/s11418-024-01803-4

The article “Ameliorative effect of bofutsushosan (Fangfengtongshengsan) extract on the progression of aging-induced obesity”, written by Takafumi Saeki, Saya Yamamoto, Junji Akaki, Takahiro Tanaka, Misaki Nakasone, Hidemasa Ikeda, Wei Wang, Makoto Inoue, Yoshiaki Manse, Kiyofumi Ninomiya and Toshio Morikawa, was originally published under exclusive license to The Author(s) under exclusive licence to The Japanese Society of Pharmacognosy. As a result of the subsequent decision to publish the article under the open access model, the article’s copyright notice was changed on 5 March 2025 to © The Author(s) 2025 and the article is now distributed under a Creative Commons Attribution 4.0 International License, which permits use, sharing, adaptation, distribution and reproduction in any medium or format, as long as you give appropriate credit to the original author(s) and the source, provide a link to the Creative Commons licence, and indicate if changes were made. The images or other third party material in this article are included in the article’s Creative Commons licence, unless indicated otherwise in a credit line to the material. If material is not included in the article’s Creative Commons licence and your intended use is not permitted by statutory regulation or exceeds the permitted use, you will need to obtain permission directly from the copyright holder. To view a copy of this licence, visit http://creativecommons.org/licenses/by/4.0.

The original article has been corrected.

