# Supplementary material for: Ameliorative effect of bofutsushosan (Fangfengtongshengsan) extract on the progression of aging-induced obesity
Source: J Nat Med. 2024 Apr 25;78(3):576–89. doi: 10.1007/s11418-024-01803-4 (PMC11937147; doi:10.1007/s11418-024-01803-4)
Supplement: Supplementary file 1 — Supplementary file1 (PDF 9883 KB) [file 11418_2024_1803_MOESM1_ESM.pdf]

# Supplementary Information

## Ameliorative effect of bofutsushosan (fangfengtongshengsan) extract on the progression of aging-induced obesity

Takafumi Saeki[1,*] ・ Saya Yamamoto[2] ・ Junji Akaki[1] ・ Takahiro Tanaka[1] ・ Misaki Nakasone[1] ・ Hidemasa Ikeda[2] ・ Wei Wang[3] ・ Makoto Inoue[3] ・ Yoshiaki Manse[2] ・ Kiyofumi Ninomiya[2,5] ・ Toshio Morikawa[2,4,*]

[1] Central R&D Laboratory, Kobayashi Pharmaceutical Co., Ltd., 1-30-3 Toyokawa, Ibaraki, Osaka 567-0057, Japan

[2] Pharmaceutical Research and Technology Institute, Kindai University, 3-4-1 Kowakae, Higashi-osaka, Osaka 577-8502, Japan

[3] Laboratory of Medicinal Resources, School of Pharmacy, Aichi Gakuin University, 1-100 Kusumoto-cho, Chikusa-ku, Nagoya 464-8650, Japan

[4] Antiaging Center, Kindai University, 3-4-1 Kowakae, Higashi-osaka, Osaka 577-8502, Japan

[5] Present address: School of Pharmacy, Shujitsu University, 1-6-1 Nishigawara, Naka-ku, Okayama 703-8516, Japan

**Figure S1.** HPLC fingerprint of the methanol extract of Japanese Angelica Root ······ S1
**Figure S2.** HPLC fingerprint of the methanol extract of Peony Root ······ S1
**Figure S3.** HPLC fingerprint of the methanol extract of Cnidium Rhizome ······ S2
**Figure S4.** HPLC fingerprint of the methanol extract of Gardenia Fruit ······ S2
**Figure S5.** HPLC fingerprint of the methanol extract of Forsythia Fruit ······ S3
**Figure S6.** HPLC fingerprint of the methanol extract of Mentha Herb ······ S3
**Figure S7.** HPLC fingerprint of the methanol extract of Ginger ······ S4
**Figure S8.** HPLC fingerprint of the methanol extract of Schizonepeta Spike ······ S4
**Figure S9.** HPLC fingerprint of the methanol extract of Saposhnikovia Root and Rhizome ······ S5
**Figure S10.** HPLC fingerprint of the methanol extract of Ephedra Herb ······ S5
**Figure S11.** HPLC fingerprint of the methanol extract of Rhubarb ······ S6
**Figure S12.** HPLC fingerprint of the methanol extract of Atractylodes Rhizome ······ S6
**Figure S13.** HPLC fingerprint of the methanol extract of Platycodon Root ······ S7
**Figure S14.** HPLC fingerprint of the methanol extract of Scutellaria Root ······ S7
**Figure S15.** HPLC fingerprint of the methanol extract of Glycyrrhiza ······ S8
**Figure S16.** Effects of glucose concentration in the medium on intracellular triglyceride content in HepG2 cells ······ S8
**Table S1.** Composition of bofutsushosan (BTS) and the origin, producing area, lot number, daily-dose, and the extraction yield of its crude drug components ······ S9
**Table S2.** Effects of 4 weeks of bofutsushosan (BTS) extract administration on the physical and biochemical parameters of mice fed a high-fat diet ······ S11
**Table S3.** Effects of two months of bofutsushosan (BTS) extract administration on the physical and biochemical parameters of mice fed a high-fat diet ······ S12
**Table S4.** Effects of crude drug extracts of each component of bofutsushosan (BTS) extract on oleic acid-albumin-induced triglyceride accumulation in HepG2 cells ······ S13
**Table S5.** Effects of crude drug extracts of each component of bofutsushosan (BTS) on triglyceride contents in high-glucose-pretreated HepG2 cells ······ S15

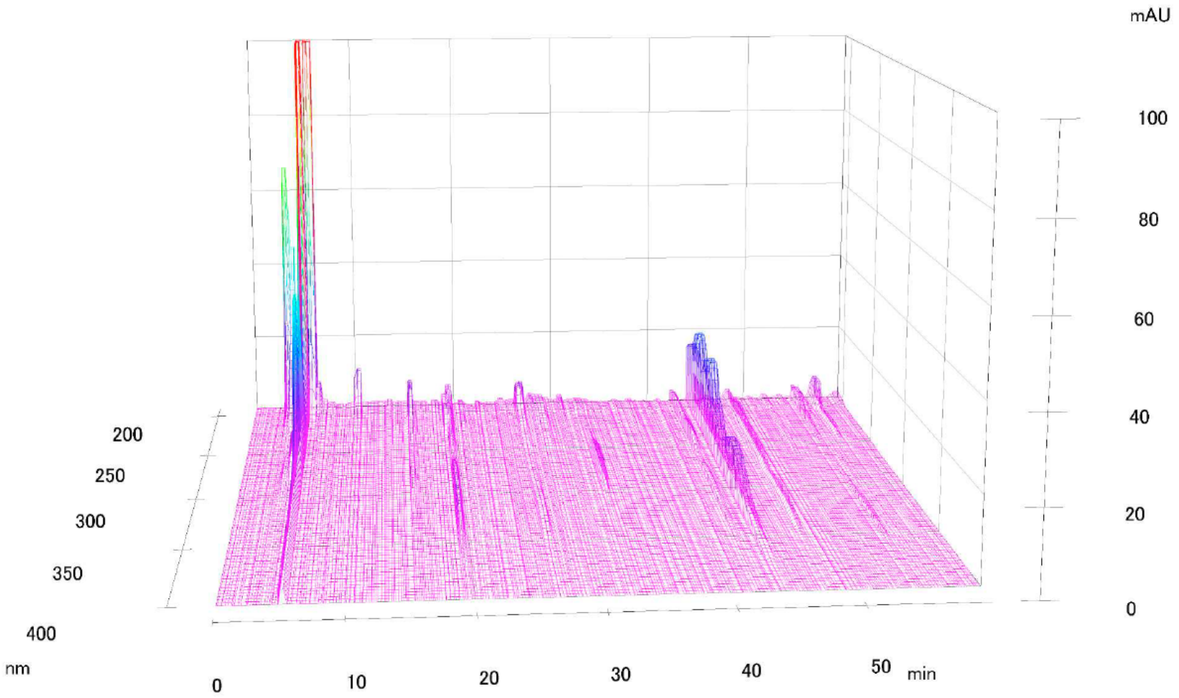


**Figure S1.** HPLC fingerprint of the methanol extract of Japanese Angelica Root

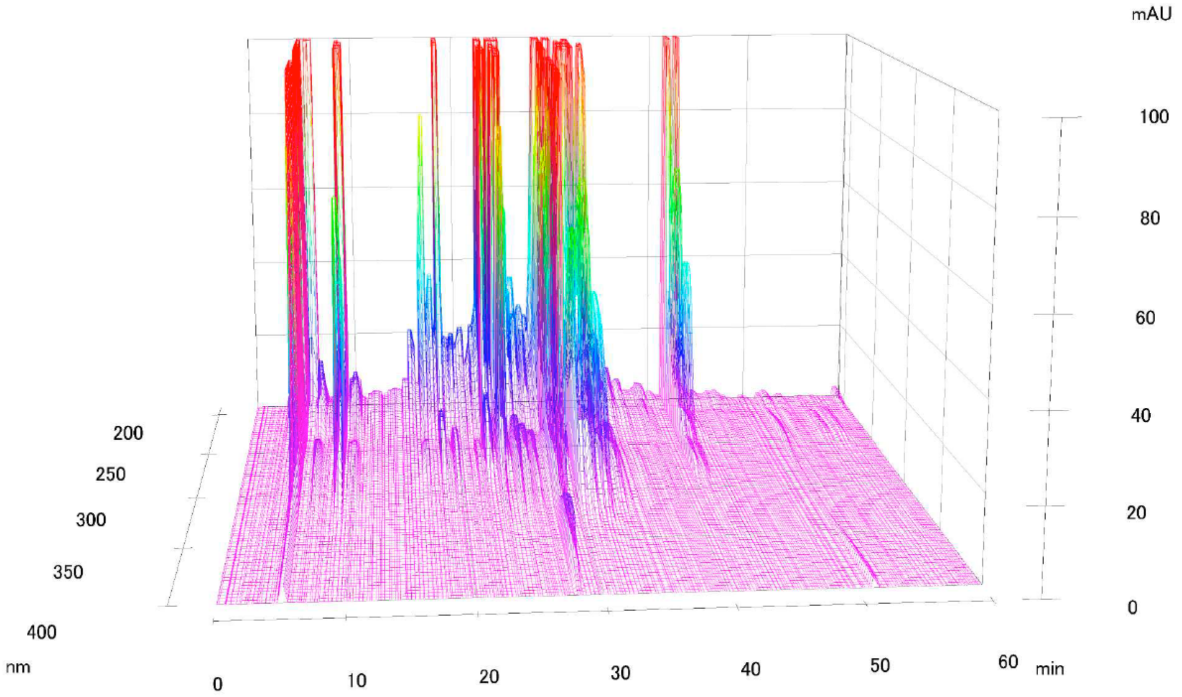


**Figure S2.** HPLC fingerprint of the methanol extract of Peony Root

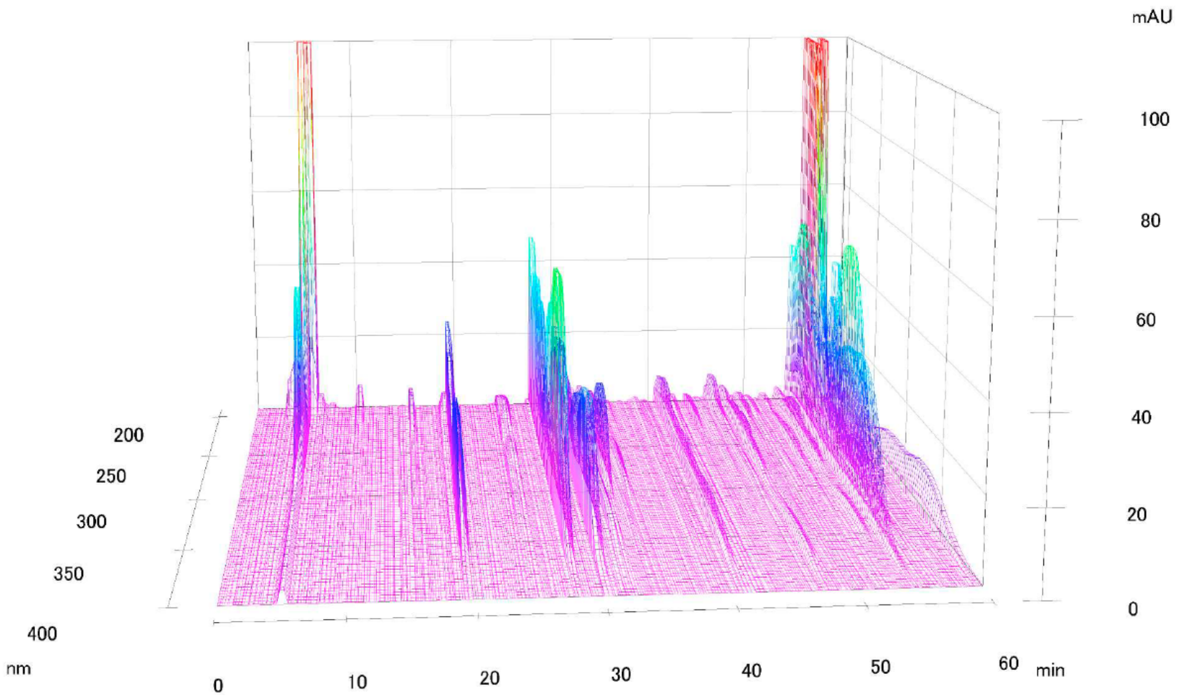

**Figure S3.** HPLC fingerprint of the methanol extract of Cnidium Rhizome

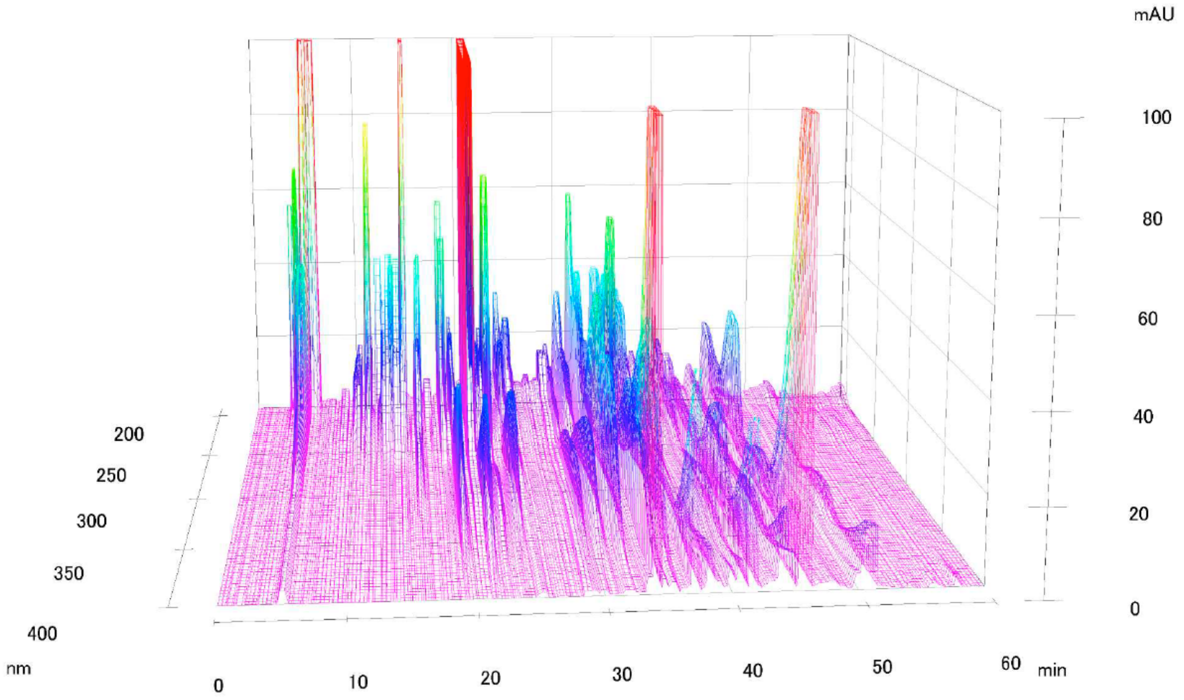

**Figure S4.** HPLC fingerprint of the methanol extract of Gardenia Fruit

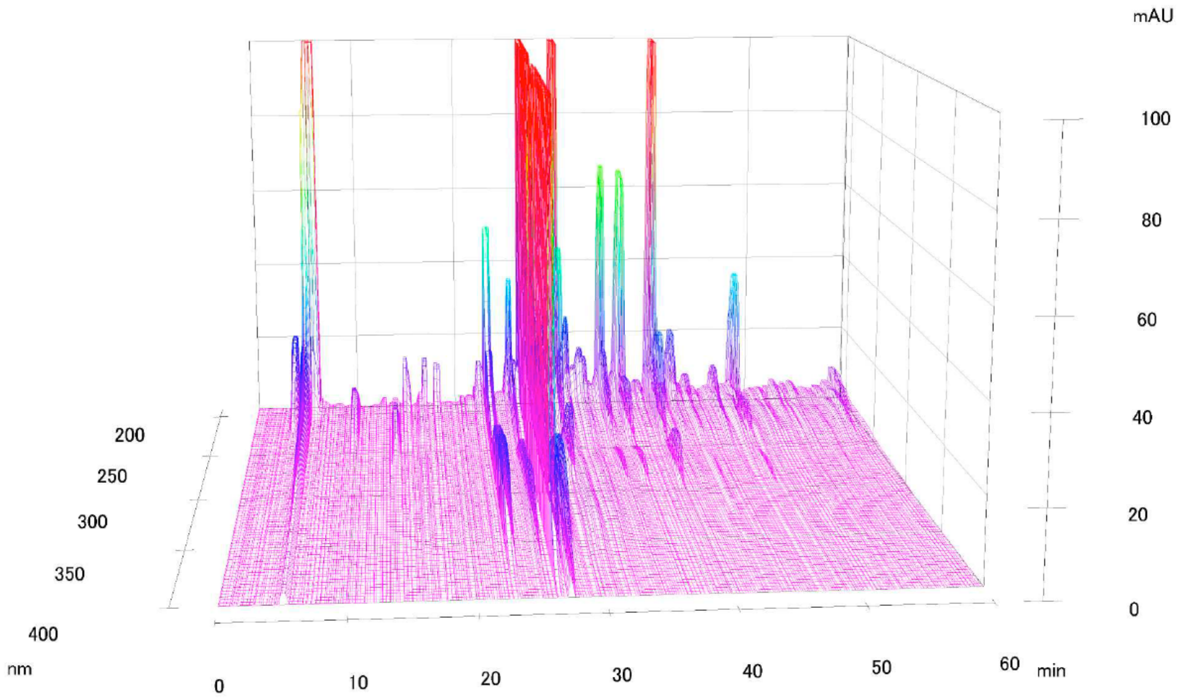


**Figure S5.** HPLC fingerprint of the methanol extract of Forsythia Fruit

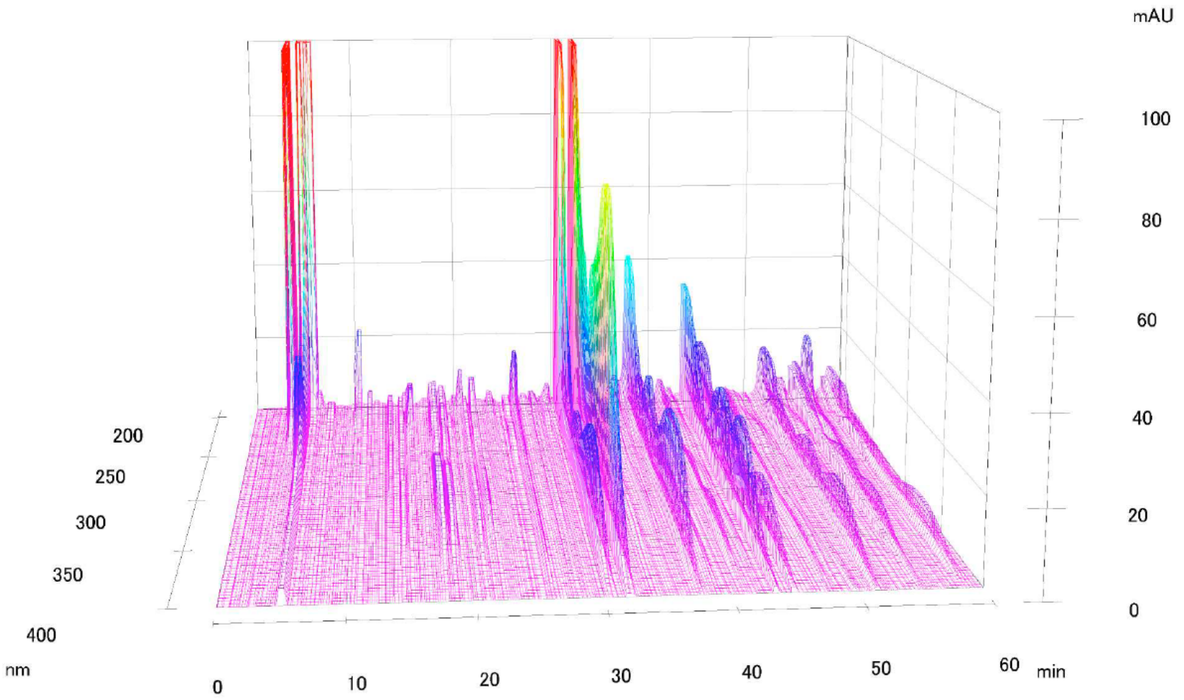


**Figure S6.** HPLC fingerprint of the methanol extract of Mentha Herb

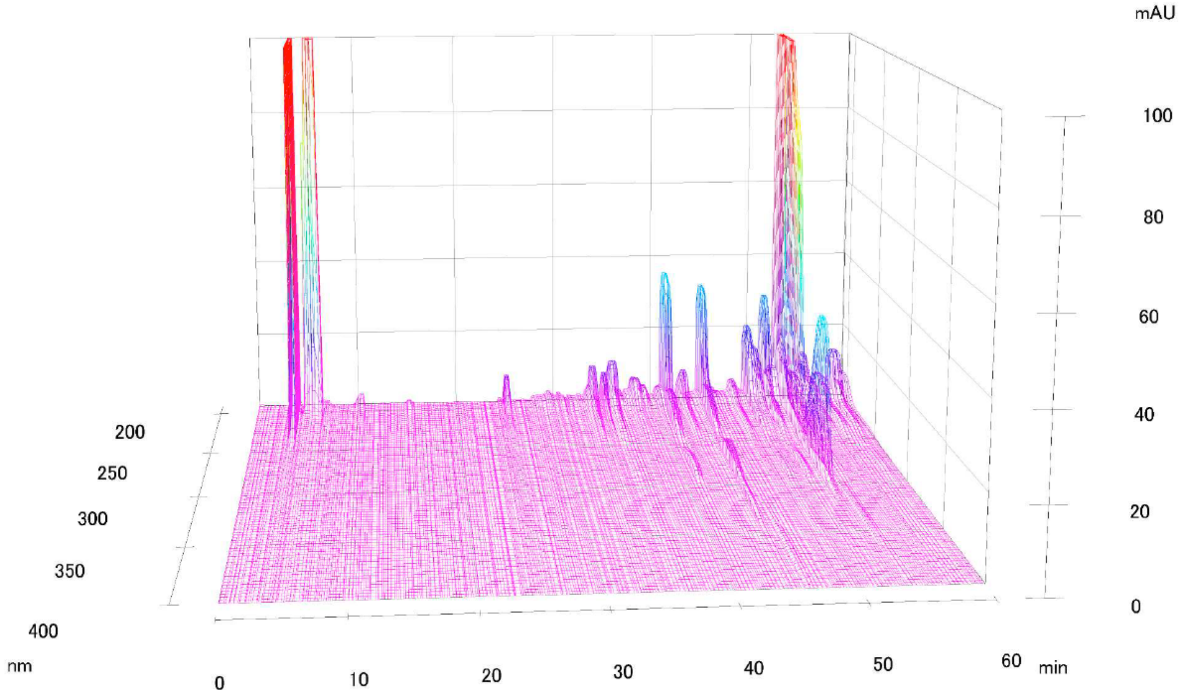


**Figure S7.** HPLC fingerprint of the methanol extract of Ginger

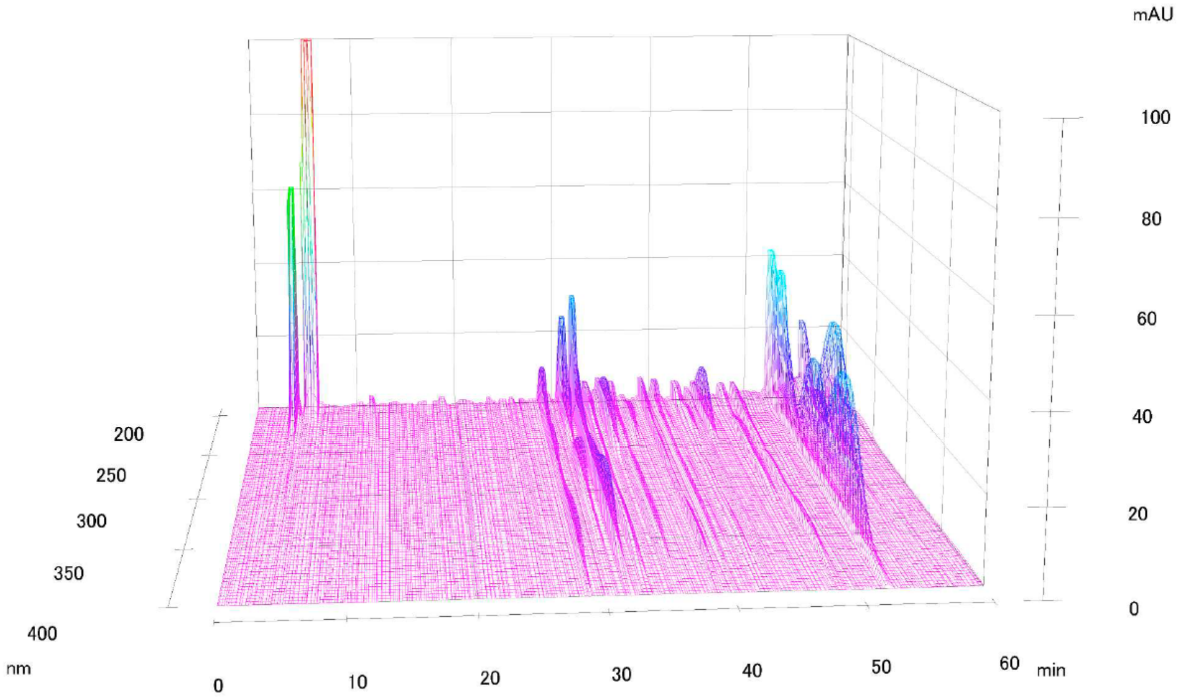


**Figure S8.** HPLC fingerprint of the methanol extract of Schizonepeta Spike

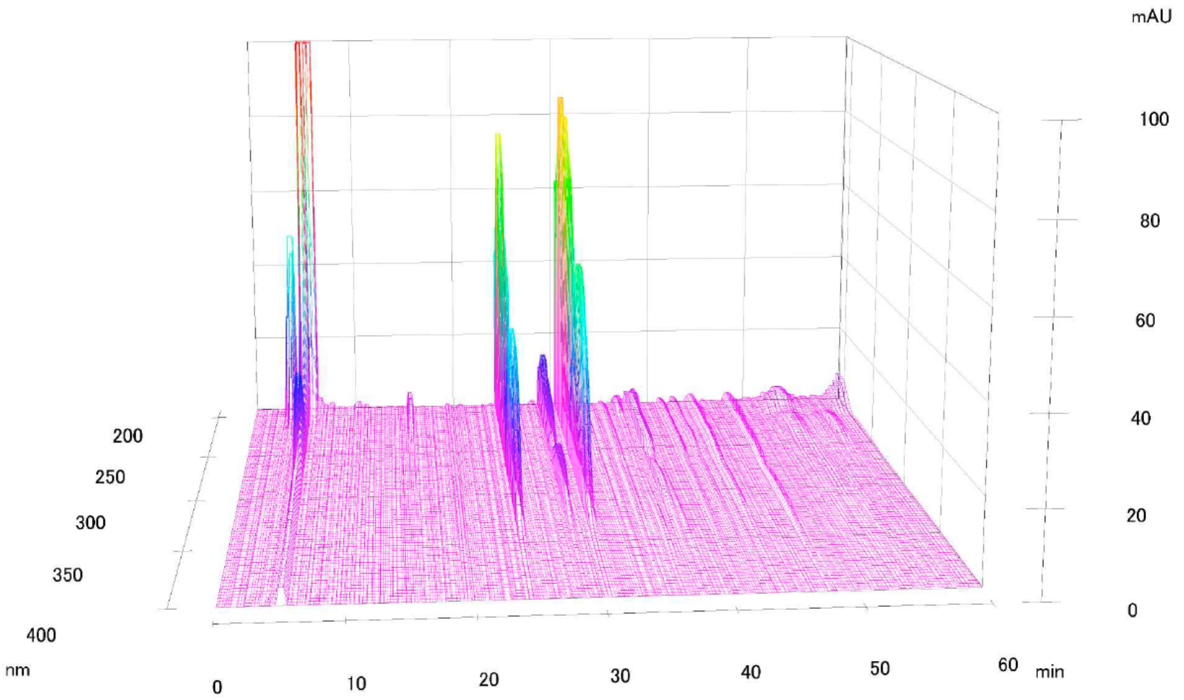


**Figure S9.** HPLC fingerprint of the methanol extract of Saposhnikovia Root and Rhizome

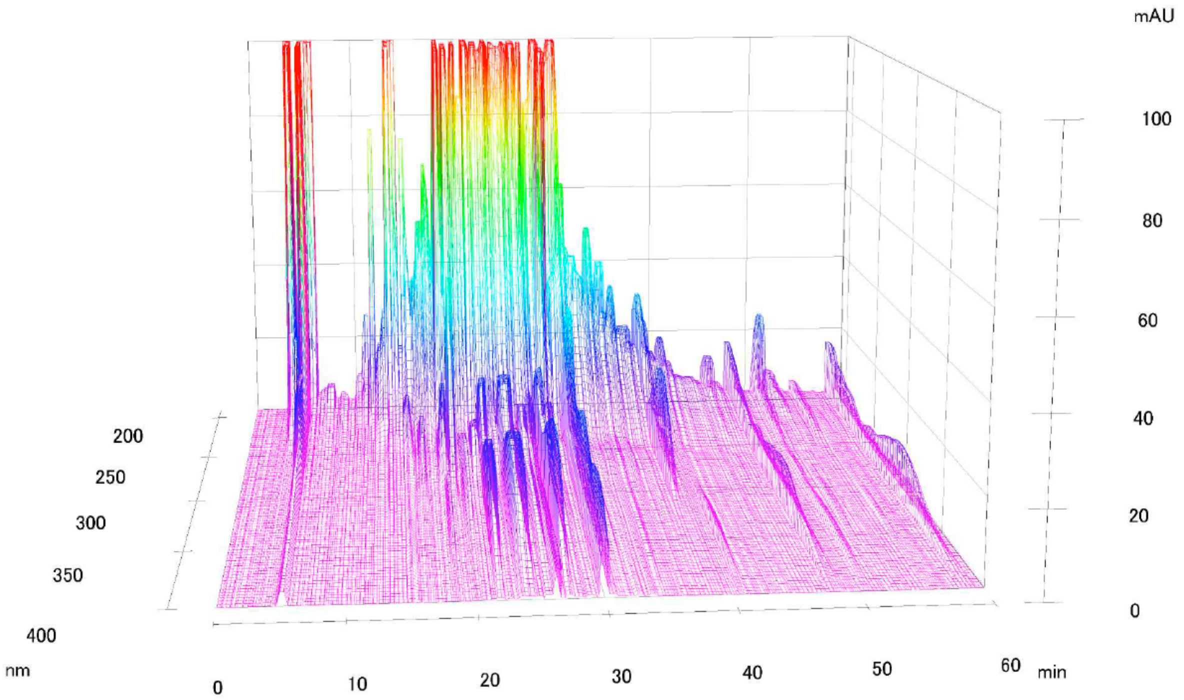


**Figure S10.** HPLC fingerprint of the methanol extract of Ephedra Herb

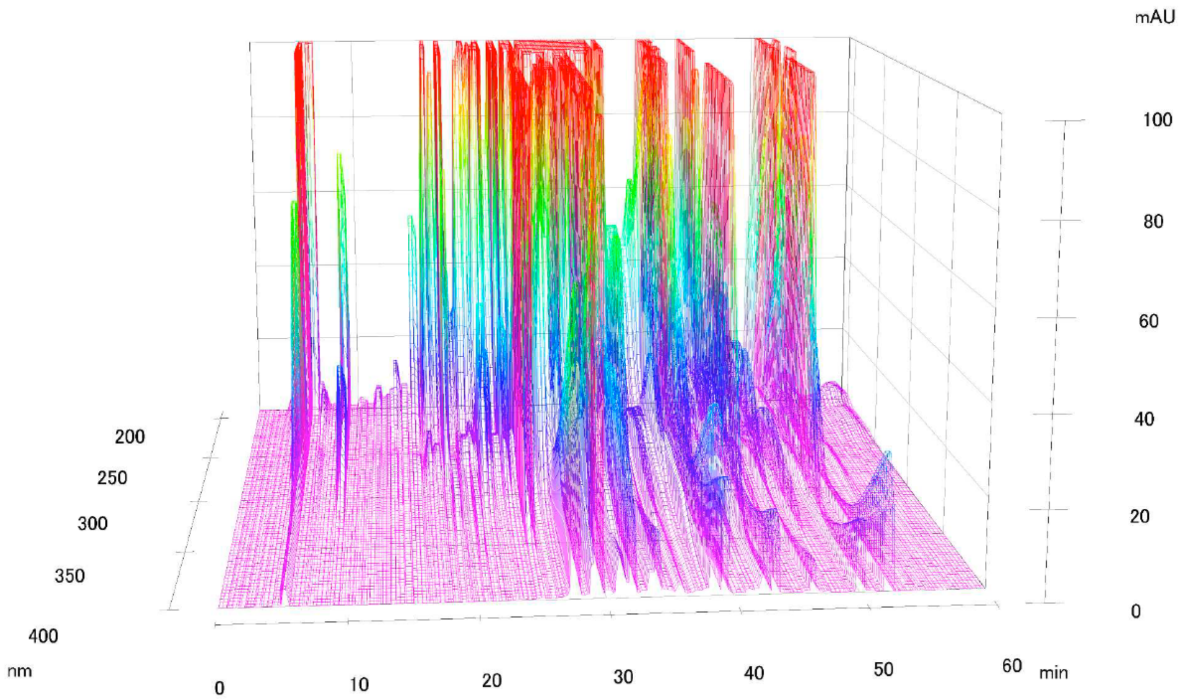


**Figure S11.** HPLC fingerprint of the methanol extract of Rhubarb

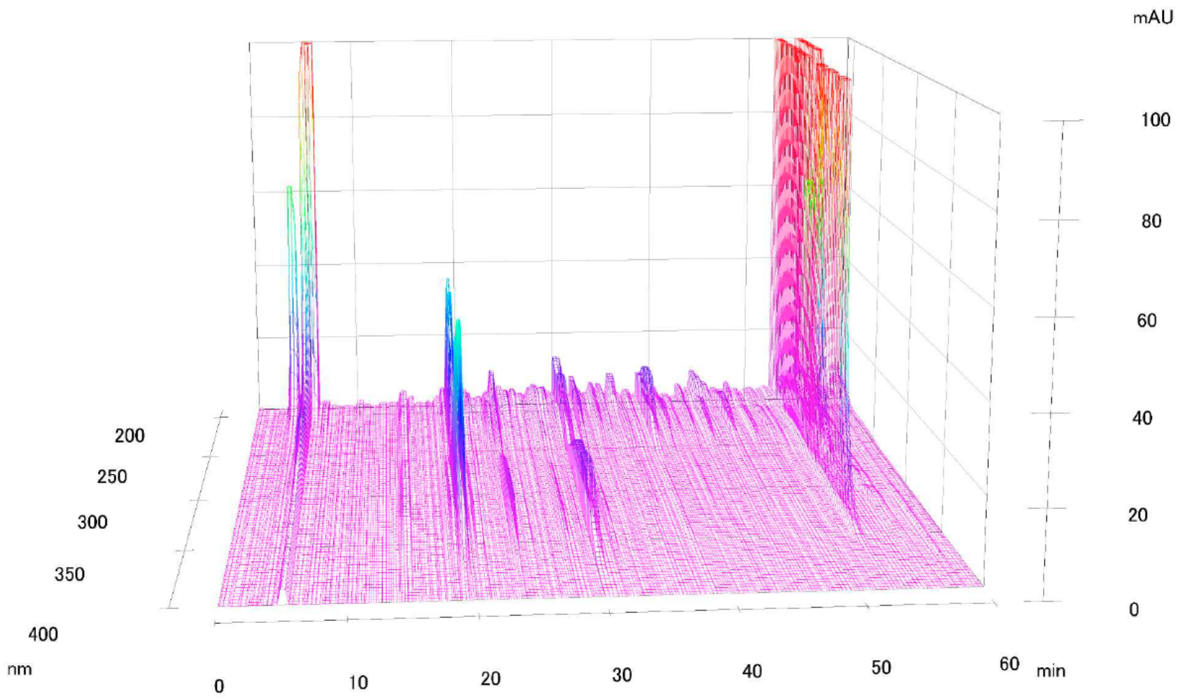


**Figure S12.** HPLC fingerprint of the methanol extract of Atractylodes Rhizome

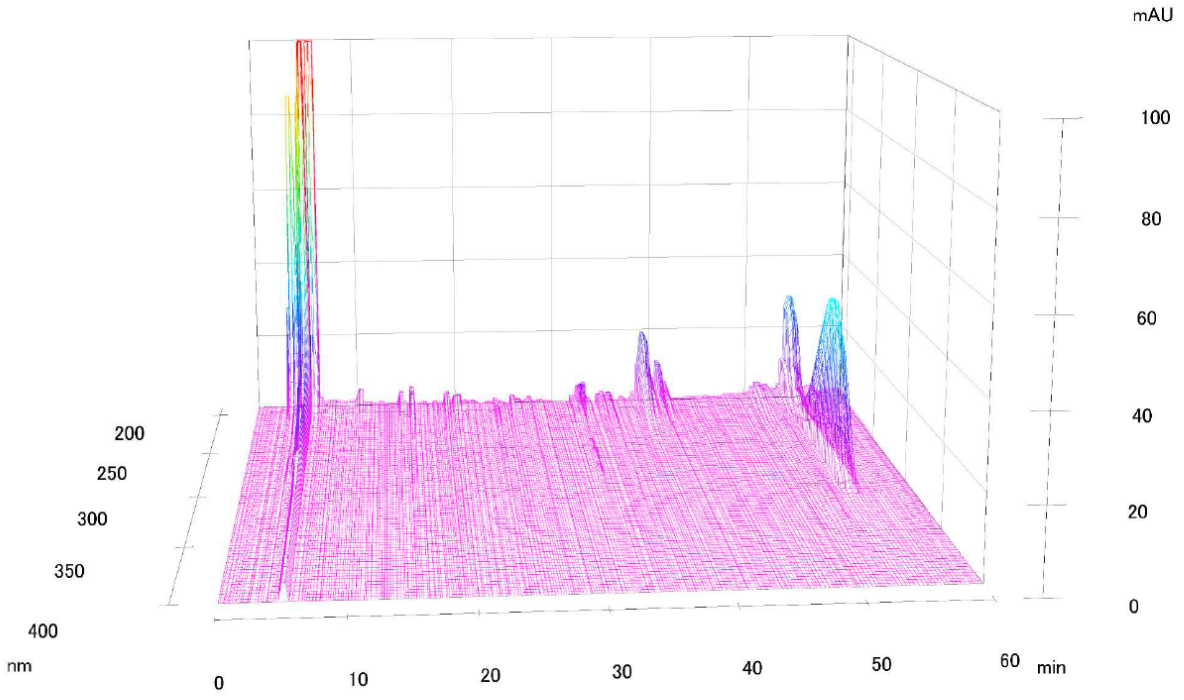


**Figure S13.** HPLC fingerprint of the methanol extract of Platycodon Root

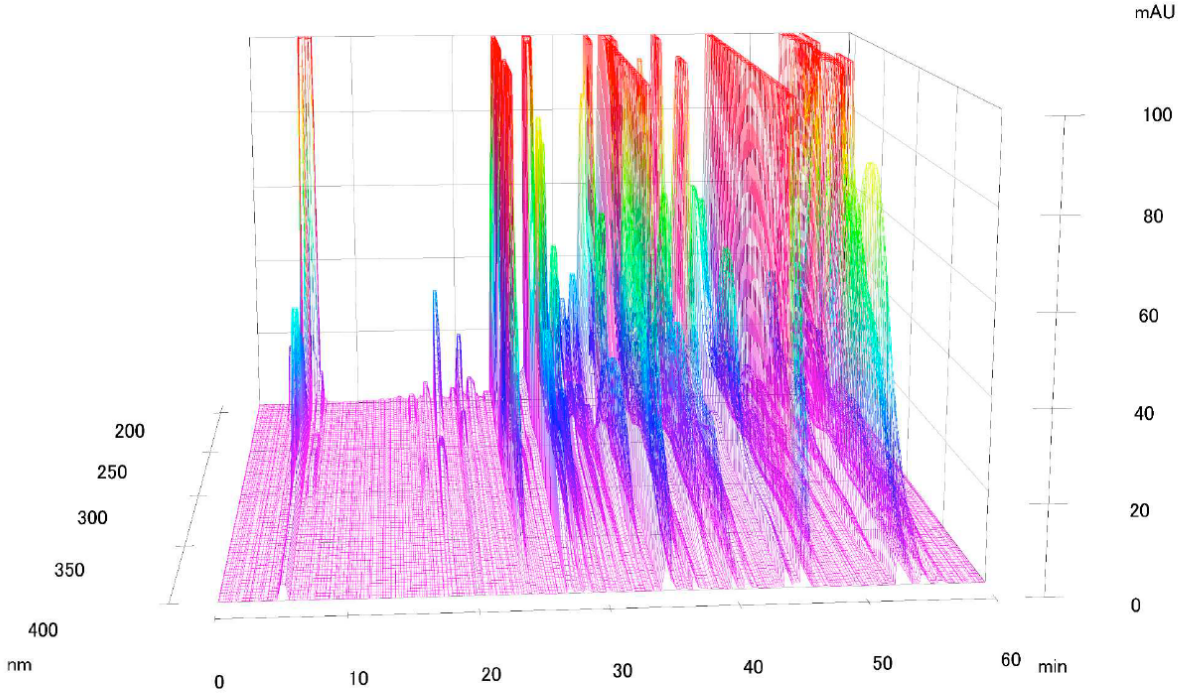


**Figure S14.** HPLC fingerprint of the methanol extract of Scutellaria Root

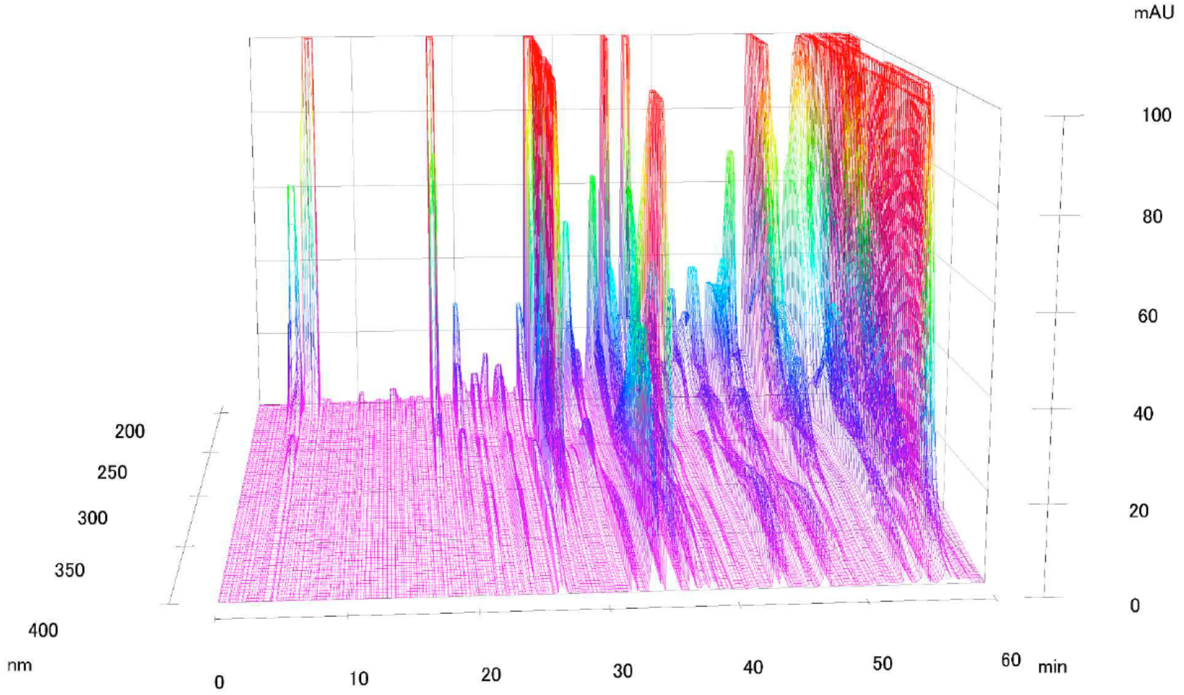


**Figure S15.** HPLC fingerprint of the methanol extract of Glycyrrhiza

Each sample solution (1 mg/mL, 10 μL) was injected to HPLC with the following conditions [column: TSK-Gel ODS-100V (4.6 × 250 mm, i.d., 5 μm); mobile phase: 10 mM aqueous phosphoric acid–$CH_3CN$ 95:5 (0 min)–40:60 (60 min), linear gradient; flow rate: 0.8 mL/min; column temperature: 40 °C; detection: 200–400 nm by a photodiode array detector].

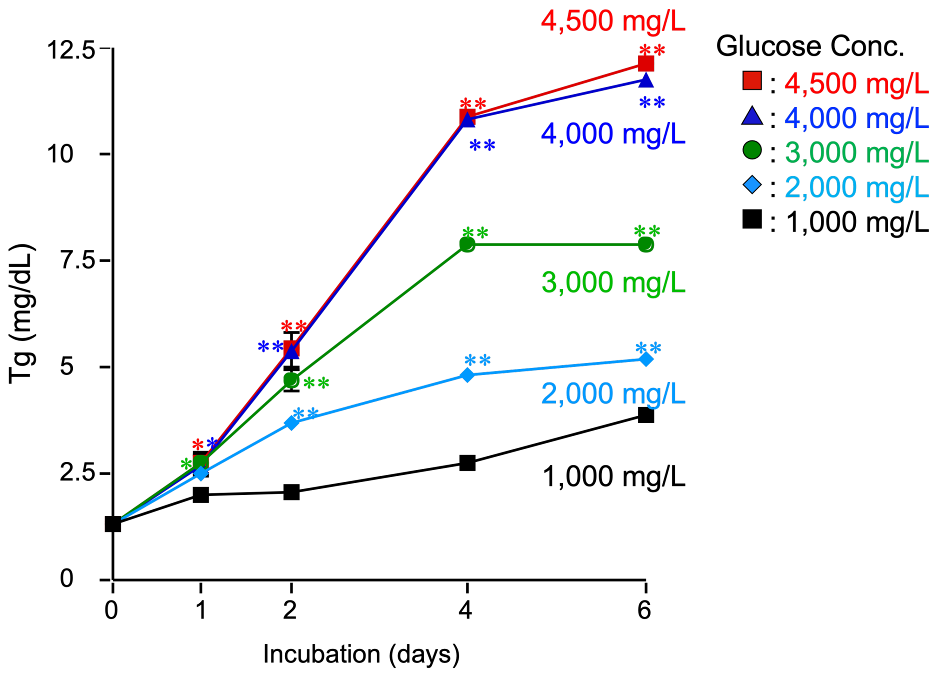


**Figure S16.** Effects of glucose concentration in the medium on intracellular triglyceride content in HepG2 cells

**Table S1.** Composition of bofutsushosan (BTS) and the origin, producing area, lot number, daily-dose, and the extraction yield of its crude drug components

| | English and Latin names of crude drug | Origin | Producing area | Lot number | Daily-dose (g) | Extraction yield (%) | |
|---|---|---|---|---|---|---|---|
| | | | | | | $H_2O$ ext. | MeOH ext. |
| 1 | Japanese Angelica Root<br>Angelicae Acutilobae Radix | The root of *Angelica acutiloba* Kitagawa | Sichuan, China | K059A6 | 1.2 | 54.3 | 3.0 |
| 2 | Peony Root<br>Paeoniae Radix | The root of *Paeonia lactiflora* Pallas | Anhui, China | K041C3 | 1.2 | 40.1 | 11.4 |
| 3 | Cnidium Rhizome<br>Cnidii Rhizoma | The rhizome of *Cnidium officinale* Makino | Japan | K048A3 | 1.2 | 38.0 | 3.9 |
| 4 | Gardenia Fruit<br>Gardeniae Fructus | The fruit of *Gardenia jasminoides* Ellis | Guangxi, China | K035C1 | 1.2 | 37.0 | 14.3 |
| 5 | Forsythia Fruit<br>Forsythiae Fructus | The fruit of *Forsythia suspensa* Vahl | Shanxi, China | K083A7 | 1.2 | 11.7 | 4.5 |
| 6 | Mentha Herb<br>Menthae Herba | The terrestrial part of *Mentha arvensis* Linné var. *piperascens* Malinvaud | Shanghai, China | K064B5 | 1.2 | 34.7 | 3.8 |
| 7 | Ginger<br>Zingiberis Rhizoma | The rhizome of *Zingiber officinale* Roscoe | Yunnan, China | K044B3 | 0.3 | 24.9 | 2.7 |
| 8 | Schizonepeta Spike<br>Schizonepetae Spica | The spike of *Nepeta tenuifolia* Briquet | Hebei, China | K024A8 | 1.2 | 18.9 | 1.5 |
| 9 | Saposhnikovia Root and Rhizome<br>Saposhnikoviae Radix | The root and rhizome of *Saposhnikovia divaricata* Schischkin | Inner Mongolia | K070B7 | 1.2 | 57.2 | 5.4 |
| 10 | Ephedra Herb<br>Ephedrae Herba | The terrestrial stem of *Ephedra sinica* Stapf | Inner Mongolia | K073B8 | 1.2 | 25.7 | 6.3 |
| 11 | Rhubarb<br>Rhei Rhizoma | The rhizome of *Rheum palmatum* Linné | Sichuan, China | K052C7 | 1.5 | 35.9 | 6.5 |
| 12 | Sodium Sulfate<br>Sal Mirabilis | Mineral substance composed of sodium sulfate | Japan | K70901 | 1.5 | 47.4 | — |
| 13 | Atractylodes Rhizome<br>Atractylodis Rhizoma | The rhizome of *Atractylodes japonica* Koidzumi ex Kitamura | Liaoning, China | K067A9 | 2.0 | 40.3 | 7.7 |
| 14 | Platycodon Root<br>Platycodi Radix | The root of *Platycodon grandiflorum* A. De Candolle | Anhui, China | K018C4 | 2.0 | 65.8 | 4.6 |
| 15 | Scutellaria Root<br>Scutellariae Radix | The root of *Scutellaria baicalensis* Georgi | Hebei, China | K008D1 | 2.0 | 42.3 | 6.0 |
| 16 | Glycyrrhiza<br>Glycyrrhizae Radix | The root and stolon of *Glycyrrhiza uralensis* Fisher | Ningxia, China | K017D3 | 2.0 | 31.2 | 12.2 |
| 17 | Gypsum<br>Gypsum Fibrosum | Natural hydrous calcium sulfate | Qinghai, China | K047B7 | 2.0 | 3.4 | — |

| | | | | | | | |
|---|---|---|---|---|---|---|---|
| 18 | Aluminium Silicate Hydrate with Silicon Dioxide<br>Kasseki | Mineral substance, mainly composed of aluminum silicate hydrate and silicon dioxide | Fijian, China | K015C2 | 3.0 | — | — |

All crude drugs met the grade standards of Japanese Pharmacopoeia 18th edition (JP XVIII).
Each crude drug (1 g) was extracted with $H_2O$ (15 mL) under boiling water for 60 min.
As for the methanol extraction, each crude drug (1 g) in methanol (15 mL) was sonicated for 30 min.
After centrifugation (3,000 rpm, 10 min), the supernatant was dried to yield the extract.

**Table S2.** Effects of 4 weeks of bofutsushosan (BTS) extract administration on the physical and biochemical parameters of mice fed a high-fat diet

| Parameter | Unit | Day | Young mice (9 weeks old) | | | Aged mice (43–63 weeks old) | | |
|---|---|---|---|---|---|---|---|---|
| | | | Normal (CE2 diet) | Control (HFD diet) | BTS (HFD diet+2% BTS) | Normal (CE2 diet) | Control (HFD diet) | BTS (HFD diet+2% BTS) |
| Body weight | g | 0 | 23.8 ± 0.5 | 23.3 ± 0.4 | 23.7 ± 0.5 | 32.9 ± 1.1 | 32.1 ± 0.7 | 31.7 ± 0.7 |
| | | 3 | 24.1 ± 0.4 | 24.8 ± 0.5 | 23.6 ± 0.4 | 33.4 ± 1.3 | 34.5 ± 0.7 | 32.1 ± 0.7$^{*}$ |
| | | 6 | 23.9 ± 0.4 | 25.0 ± 0.3 | 23.8 ± 0.4 | 32.5 ± 1.3 | 35.7 ± 0.7$^{\#}$ | 32.8 ± 0.7$^{*}$ |
| | | 9 | 24.4 ± 0.5 | 25.2 ± 0.3 | 24.5 ± 0.5 | 32.6 ± 1.3 | 36.4 ± 0.8$^{\#}$ | 32.9 ± 0.8$^{*}$ |
| | | 13 | 24.5 ± 0.4 | 25.8 ± 0.3$^{\#}$ | 24.9 ± 0.5 | 31.9 ± 1.4 | 37.4 ± 0.8$^{\#\#}$ | 33.7 ± 1.0$^{*}$ |
| | | 17 | 24.8 ± 0.4 | 26.1 ± 0.2$^{\#}$ | 24.9 ± 0.6 | 32.2 ± 1.5 | 38.6 ± 1.0$^{\#\#}$ | 34.3 ± 0.9$^{**}$ |
| | | 20 | 24.7 ± 0.3 | 27.0 ± 0.3$^{\#\#}$ | 25.4 ± 0.6$^{*}$ | 32.2 ± 1.2 | 39.3 ± 0.9$^{\#\#}$ | 34.7 ± 0.9$^{**}$ |
| | | 24 | 25.2 ± 0.4 | 27.5 ± 0.4$^{\#\#}$ | 25.7 ± 0.5$^{*}$ | 33.0 ± 1.4 | 40.2 ± 1.0$^{\#\#}$ | 35.0 ± 1.0$^{**}$ |
| | | 28 | 25.3 ± 0.4 | 28.2 ± 0.4$^{\#\#}$ | 25.8 ± 0.4$^{**}$ | 32.8 ± 1.5 | 40.8 ± 1.1$^{\#\#}$ | 35.2 ± 1.0$^{**}$ |
| Body weight gain | g | | 1.6 ± 0.2 | 4.9 ± 0.3$^{\#\#}$ | 2.1 ± 0.1$^{**}$ | -0.1 ± 0.6 | 8.7 ± 0.5$^{\#\#}$ | 3.5 ± 0.7$^{**}$ |
| Food intake | g/day | | 4.8 ± 0.0 | 2.7 ± 0.0 | 2.6 ± 0.0$^{*}$ | 4.2 ± 0.1 | 3.3 ± 0.1$^{\#\#}$ | 3.0 ± 0.1$^{*}$ |
| | g/kg bw/day | | 197.0 ± 3.8 | 103.7 ± 1.1$^{\#\#}$ | 103.4 ± 1.8 | 129.4 ± 4.0 | 89.3 ± 1.7$^{\#\#}$ | 88.1 ± 0.9 |
| | kcal/kg bw/day | | 668.1 ± 12.7 | 526.5 ± 5.3$^{\#\#}$ | 514.8 ± 9.1 | 438.7 ± 13.6 | 453.3 ± 8.7 | 438.5 ± 4.3 |
| Visceral fat | g | 24 | 0.39 ± 0.05 | 1.22 ± 0.04$^{\#\#}$ | 0.70 ± 0.15$^{**}$ | 1.20 ± 0.28 | 4.02 ± 0.25$^{\#\#}$ | 2.71 ± 0.21$^{**}$ |
| Subcutaneous fat | g | 24 | 0.22 ± 0.02 | 0.73 ± 0.05$^{\#\#}$ | 0.47 ± 0.09$^{*}$ | 0.75 ± 0.15 | 2.42 ± 0.13$^{\#\#}$ | 1.63 ± 0.14$^{**}$ |
| Total fat | g | 24 | 0.61 ± 0.07 | 1.95 ± 0.08$^{\#\#}$ | 1.17 ± 0.23$^{**}$ | 1.96 ± 0.42 | 6.44 ± 0.37$^{\#\#}$ | 4.34 ± 0.35$^{**}$ |
| Liver weight | g | 28 | 1.38 ± 0.03 | 1.27 ± 0.03$^{\#}$ | 1.23 ± 0.03 | 1.62 ± 0.09 | 1.98 ± 0.10$^{\#}$ | 1.64 ± 0.08$^{*}$ |
| Epididymal WAT | g | 28 | 0.43 ± 0.01 | 0.94 ± 0.03$^{\#\#}$ | 0.60 ± 0.07$^{**}$ | 0.95 ± 0.17 | 2.53 ± 0.20$^{\#\#}$ | 1.75 ± 0.11$^{**}$ |
| Interscapular BAT | mg | 28 | 148.6 ± 14.9 | 150.2 ± 17.2 | 124.6 ± 13.2 | 185.0 ± 15.9 | 238.3 ± 22.0 | 188.0 ± 14.0 |
| GLU | mg/dL | 28 | 170.4 ± 3.3 | 224.0 ± 7.9$^{\#\#}$ | 210.8 ± 13.6 | 176.4 ± 12.8 | 222.1 ± 16.3 | 206.7 ± 9.7 |
| TG | mg/dL | 28 | 64.0 ± 17.1 | 140.7 ± 34.6 | 74.8 ± 7.2 | 109.0 ± 23.4 | 64.4 ± 9.4 | 72.8 ± 9.2 |
| NEFA | µEq/L | 28 | 607.4 ± 71.6 | 964.7 ± 146.6 | 863.6 ± 62.8 | 711.8 ± 47.7 | 724.3 ± 48.9 | 744.5 ± 73.0 |
| T-CHO | mg/dL | 28 | 70.4 ± 3.9 | 187.2 ± 5.5$^{\#\#}$ | 180.6 ± 8.4 | 70.8 ± 2.4 | 197.7 ± 3.0$^{\#\#}$ | 161.8 ± 9.0$^{**}$ |
| AST | IU/L | 28 | 32.4 ± 1.2 | 33.3 ± 1.5 | 33.0 ± 1.8 | 38.4 ± 2.7 | 70.1 ± 8.6$^{\#}$ | 52.0 ± 6.5 |
| ALT | IU/L | 28 | 21.0 ± 1.3 | 13.8 ± 1.3$^{\#\#}$ | 12.4 ± 0.8 | 27.0 ± 3.6 | 33.9 ± 6.4 | 21.3 ± 2.7 |
| ALP | IU/L | 28 | 292.6 ± 4.3 | 227.5 ± 8.3$^{\#\#}$ | 242.4 ± 10.7 | 197.8 ± 8.2 | 136.1 ± 5.4$^{\#\#}$ | 132.2 ± 6.6 |

Mean ± S.E. (n = 5–8).

$^{\#}p < 0.05$, $^{\#\#}p < 0.01$ *vs.* Normal, $^{*}p < 0.05$, $^{**}p < 0.01$ *vs.* Control (*t*-test).

**Table S3.** Effects of two months of bofutsushosan (BTS) extract administration on the physical and biochemical parameters of mice fed a high-fat diet

| Parameter | Unit | Day | Young mice (9 weeks old) | | Aged mice (64–65 weeks old) | |
|---|---|---|---|---|---|---|
| | | | Control (HFD diet) | BTS (HFD diet+2% BTS) | Control (HFD diet) | BTS (HFD diet+2% BTS) |
| Body weight | g | 0 | 22.3 ± 0.2 | 22.3 ± 0.2 | 34.9 ± 0.9 | 34.2 ± 0.8 |
| | | 3 | 24.2 ± 0.4 | 22.2 ± 0.1** | 38.4 ± 1.0 | 34.8 ± 0.7* |
| | | 7 | 24.5 ± 0.5 | 23.1 ± 0.3* | 39.7 ± 1.2 | 35.6 ± 0.8* |
| | | 13 | 25.5 ± 0.4 | 24.0 ± 0.2* | 42.1 ± 1.1 | 36.6 ± 0.9** |
| | | 20 | 26.7 ± 0.6 | 25.1 ± 0.4* | 43.8 ± 1.3 | 37.4 ± 1.1** |
| | | 28 | 28.0 ± 0.7 | 25.9 ± 0.4* | 45.7 ± 1.4 | 38.1 ± 1.1** |
| | | 34 | 29.4 ± 0.8 | 26.5 ± 0.3** | 46.5 ± 1.4 | 39.0 ± 1.2** |
| | | 42 | 30.5 ± 0.7 | 27.5 ± 0.5** | 47.0 ± 1.6 | 39.8 ± 1.3** |
| | | 49 | 31.7 ± 0.8 | 28.6 ± 0.5** | 47.7 ± 1.6 | 40.5 ± 1.4** |
| | | 57 | 32.6 ± 1.0 | 29.5 ± 0.6* | 48.2 ± 1.6 | 40.6 ± 1.3** |
| | | 62 | 33.4 ± 0.9 | 30.2 ± 0.6* | 49.0 ± 1.5 | 41.3 ± 1.4** |
| Body weight gain | g | | 11.1 ± 0.9 | 7.9 ± 0.7* | 14.1 ± 0.9 | 7.1 ± 1.0** |
| Food intake | g/day | | 2.7 ± 0.1 | 2.7 ± 0.1 | 3.5 ± 0.1 | 3.2 ± 0.1 |
| | g/kg bw/day | | 96.8 ± 2.0 | 102.5 ± 2.4 | 79.5 ± 1.2 | 84.4 ± 0.8* |
| | kcal/kg bw/day | | 491.5 ± 10.0 | 510.0 ± 12.0 | 403.5 ± 6.3 | 419.9 ± 4.2 |
| Liver weight | g | 62 | 1.50 ± 0.07 | 1.43 ± 0.03 | 2.99 ± 0.28 | 2.21 ± 0.13* |
| Liver TG | mg/g protein | 62 | 163.4 ± 11.1 | 115.1 ± 10.9** | 264.2 ± 24.6 | 187.1 ± 40.9 |
| AST | IU/L | 62 | 38.7 ± 1.7 | 43.0 ± 4.5 | 152.4 ± 22.0 | 70.0 ± 4.9** |
| ALT | IU/L | 62 | 15.1 ± 1.2 | 16.0 ± 0.7 | 97.8 ± 23.1 | 35.3 ± 4.3* |
| ALP | IU/L | 62 | 207.4 ± 3.8 | 209.6 ± 6.0 | 217.3 ± 19.8 | 150.7 ± 6.7* |

Mean ± S.E. (n = 5–8).

$^{*}p < 0.05$, $^{**}p < 0.01$ *vs.* Control (*t*-test).

**Table S4.** Effects of crude drug extracts of each component of bofutsushosan (BTS) extract on oleic acid-albumin-induced triglyceride accumulation in HepG2 cells

| Treatment | TG/protein (% of control) | | | | |
|---|---|---|---|---|---|
| 5% Oleic acid-albumin | – | + | + | + | + |
| Conc. ($\mu$g/mL) | 0 | 0 | 10 | 30 | 100 |
| BTS ext. | 40.1 ± 1.1** | 100.0 ± 1.2 | 97.2 ± 1.6 | 95.3 ± 2.3 | 89.1 ± 1.6** |
| | (82.2 ± 0.8**) | (100.0 ± 1.0) | (104.1 ± 1.0) | (105.5 ± 1.0) | (107.2 ± 1.5) |
| $H_2O$ extract | | | | | |
| 1. Japanese Angelica Root | 44.7 ± 0.7** | 100.0 ± 1.2 | 101.5 ± 2.2 | 101.3 ± 1.3 | 98.2 ± 1.5 |
| | (81.8 ± 0.9**) | (100.0 ± 1.4) | (98.6 ± 2.9) | (101.0 ± 1.8) | (103.9 ± 1.9) |
| 2. Peony Root | 41.0 ± 0.7** | 100.0 ± 2.9 | 100.4 ± 1.0 | 98.3 ± 1.0 | 93.2 ± 1.1 |
| | (84.8 ± 1.9**) | (100.0± 3.0) | (98.6 ± 0.8) | (99.4 ± 1.1) | (100.4 ± 1.6) |
| 3. Cnidium Rhizome | 39.1 ± 1.4** | 100.0 ± 2.3 | 100.5 ± 3.5 | 97.1 ± 2.3 | 98.8 ± 1.7 |
| | (80.5 ± 0.5**) | (100.0 ± 0.7) | (99.1 ± 2.6) | (106.3 ± 1.6) | (104.8 ± 2.9) |
| 4. Gardenia Fruit | 38.0 ± 1.9** | 100.0 ± 1.5 | 104.0 ± 3.4 | 97.9 ± 3.1 | 100.1 ± 2.5 |
| | (80.6 ± 0.8**) | (100.0 ± 1.4) | (97.0 ± 1.4) | (100.4 ± 2.5) | (100.5 ± 0.3) |
| 5. Forsythia Fruit | 46.6 ± 2.4** | 100.0 ± 2.1 | 105.0 ± 0.9 | 105.7 ± 4.2 | 88.9 ± 4.4** |
| | (82.1 ± 1.6**) | (100.0 ± 1.7) | (96.9 ± 1.9) | (95.6 ± 3.2) | (101.7 ± 4.1) |
| 6. Mentha Herb | 41.5 ± 2.2** | 100.0 ± 1.2 | 98.5 ± 2.5 | 91.2 ± 2.1* | 88.6 ± 1.4** |
| | (86.8 ± 2.0**) | (100.0 ± 1.8) | (101.4 ± 1.4) | (104.2 ± 2.4) | (101.1 ± 0.3) |
| 7. Ginger | 46.0 ± 1.4** | 100.0 ± 1.8 | 95.6 ± 1.4 | 99.0 ± 2.9 | 103.3 ± 2.1 |
| | (87.7 ± 1.2**) | (100.0 ± 1.4) | (98.5 ± 0.4) | (97.1 ± 2.4) | (98.4 ± 1.3) |
| 8. Schizonepeta Spike | 44.7 ± 0.5** | 100.0 ± 2.2 | 93.3 ± 1.3 | 91.4 ± 1.7* | 86.9 ± 3.0** |
| | (81.6 ± 0.5**) | (100.0 ± 2.2) | (100.7 ± 1.1) | (104.5 ± 1.9) | (106.3 ± 3.1) |
| 9. Saposhnikovia Root and Rhizome | 42.8 ± 1.1** | 100.0 ± 2.4 | 96.3 ± 1.4 | 103.8 ± 4.4 | 107.2 ± 1.3 |
| | (80.3 ± 0.6**) | (100.0 ± 1.8) | (98.2 ± 0.7) | (96.1 ± 2.9) | (93.3 ± 1.5#) |
| 10. Ephedra Herb | 39.9 ± 2.2** | 100.0 ± 2.1 | 102.5 ± 2.6 | 102.2 ± 2.5 | 98.2 ± 1.1 |
| | (80.6 ± 0.6**) | (100.0 ± 1.6) | (98.8 ± 1.7) | (104.0 ± 2.0) | (105.5 ± 0.8) |
| 11. Rhubarb | 26.8 ± 1.7** | 100.0 ± 3.0 | 97.3 ± 2.2 | 89.4 ± 0.7** | 89.5 ± 1.6** |
| | (82.1 ± 0.9**) | (100.0 ± 1.6) | (95.9 ± 1.9) | (101.2 ± 0.9) | (101.5 ± 0.9) |
| 12. Sodium Sulfate | 53.2 ± 1.8** | 100.0 ± 2.2 | 99.3 ± 1.7 | 100.8 ± 1.9 | 112.8 ± 2.4 |
| | (85.7 ± 2.1**) | (100.0 ± 2.3) | (100.5 ± 1.0) | (101.2 ± 1.6) | (91.2 ± 1.1##) |
| 13. Atractylodes Rhizome | 35.4 ± 1.5** | 100.0 ± 2.6 | 108.2 ± 1.6 | 114.2 ± 4.1* | 115.5 ± 3.6 |
| | (79.0 ± 0.9**) | (100.0 ± 1.1) | (96.4 ± 1.6) | (94.9 ± 1.5) | (95.5 ± 1.9) |
| 14. Platycodon Root | 35.7 ± 2.4** | 100.0 ± 1.8 | 95.0 ± 2.2 | 96.5 ± 1.7 | 119.3 ± 2.5 |
| | (75.8 ± 1.8**) | (100.0 ± 1.4) | (97.0 ± 1.4) | (103.0 ± 0.9) | (102.8 ± 1.2) |
| 15. Scutellaria Root | 37.9 ± 2.0** | 100.0 ± 2.5 | 97.8 ± 4.2 | 97.1 ± 4.0 | 85.3 ± 3.8** |
| | (81.4 ± 1.4**) | (100.0 ± 2.8) | (100.6 ± 1.8) | (102.2 ± 3.9) | (104.5 ± 0.9) |

| | | | | | |
|---|---|---|---|---|---|
| 16. Glycyrrhiza | 56.5 ± 2.9$^{**}$ | 100.0 ± 2.1 | 110.1 ± 3.4 | 101.7 ± 0.5 | 103.2 ± 1.6 |
| | (70.8 ± 1.1$^{**}$) | (100.0 ± 1.5) | (89.7 ± 1.7) | (96.2 ± 0.6) | (93.2 ± 0.6) |
| MeOH extract | | | | | |
| 1. Japanese Angelica Root | 42.9 ± 2.2$^{**}$ | 100.0 ± 0.9 | 100.7 ± 0.9 | 99.0 ± 0.6 | 90.2 ± 2.5$^{**}$ |
| | (81.7 ± 1.1$^{**}$) | (100.0 ± 2.5) | (99.6 ± 1.9) | (100.7 ± 2.0) | (107.1 ± 3.3) |
| 2. Peony Root | 44.9 ± 2.3$^{**}$ | 100.0 ± 3.7 | 99.6 ± 2.4 | 100.8 ± 0.5 | 101.7 ± 3.7 |
| | (87.8 ± 2.4$^{**}$) | (100.0 ± 0.7) | (98.0 ± 0.3) | (99.5 ± 1.8) | (95.9 ± 4.3) |
| 3. Cnidium Rhizome | 40.4 ± 0.6$^{**}$ | 100.0 ± 2.2 | 99.5 ± 1.5 | 96.6 ± 1.0 | 96.9 ± 1.3 |
| | (84.2 ± 1.5$^{**}$) | (100.0 ± 2.3) | (101.7 ± 2.5) | (103.7 ± 1.3) | (107.8 ± 2.3) |
| 4. Gardenia Fruit | 41.8 ± 1.7$^{**}$ | 100.0 ± 2.0 | 97.3 ± 1.3 | 96.4 ± 1.3 | 94.8 ± 1.4 |
| | (80.4 ± 0.8$^{**}$) | (100.0 ± 2.0) | (101.0 ± 1.2) | (102.6 ± 2.6) | (99.6 ± 1.2) |
| 5. Forsythia Fruit | 43.7 ± 0.6$^{**}$ | 100.0 ± 0.5 | 81.7 ± 1.6$^{*}$ | 59.0 ± 3.0$^{**}$ | 131.4 ± 4.2$^{**}$ |
| | (85.9 ± 2.3$^{**}$) | (100.0 ± 2.3) | (103.5 ± 1.1) | (103.0 ± 2.7) | (68.0 ± 1.0$^{\#\#}$) |
| 6. Mentha Herb | 40.9 ± 1.4$^{**}$ | 100.0 ± 1.8 | 92.3 ± 1.3 | 94.7 ± 1.0 | 83.5 ± 3.0$^{**}$ |
| | (82.1 ± 1.0$^{**}$) | (100.0 ± 1.2) | (101.7 ± 0.8) | (94.6 ± 1.3) | (99.3 ± 1.8) |
| 7. Ginger | 43.8 ± 0.6$^{**}$ | 100.0 ± 2.8 | 98.4 ± 1.9 | 113.1 ± 1.4 | 162.8 ± 3.7$^{**}$ |
| | (91.1 ± 1.5$^{**}$) | (100.0 ± 1.9) | (103.8 ± 1.4) | (102.1 ± 1.3) | (98.0 ± 2.0) |
| 8. Schizonepeta Spike | 38.6 ± 0.8$^{**}$ | 100.0 ± 4.0 | 91.6 ± 0.5$^{*}$ | 80.4 ± 1.2$^{**}$ | 63.4 ± 1.0$^{**}$ |
| | (89.3 ± 0.9$^{**}$) | (100.0 ± 2.0) | (105.4 ± 1.4) | (109.8 ± 1.6) | (113.6 ± 1.7$^{\#\#}$) |
| 9. Saposhnikovia Root and Rhizome | 42.2 ± 2.2$^{**}$ | 100.0 ± 2.3 | 103.1 ± 1.4 | 100.6 ± 1.4 | 99.0 ± 3.6 |
| | (80.4 ± 1.7$^{**}$) | (100.0 ± 2.1) | (93.9 ± 0.7) | (96.6 ± 1.0) | (95.9 ± 0.1) |
| 10. Ephedra Herb | 42.7 ± 1.8$^{**}$ | 100.0 ± 1.1 | 99.5 ± 2.3 | 106.1 ± 2.1 | 107.0 ± 1.9 |
| | (78.8 ± 0.6$^{**}$) | (100.0 ± 1.1) | (100.8 ± 1.5) | (100.8 ± 1.2) | (102.9 ± 1.9) |
| 11. Rhubarb | 31.5 ± 2.9$^{**}$ | 100.0 ± 1.3 | 94.1 ± 1.1 | 88.2 ± 4.3$^{**}$ | 116.6 ± 3.6$^{*}$ |
| | (84.4 ± 0.6$^{**}$) | (100.0 ± 0.7) | (103.6 ± 1.9) | (110.2 ± 3.3) | (99.5 ± 2.0) |
| 13. Atractylodes Rhizome | 37.3 ± 0.5$^{**}$ | 100.0 ± 2.0 | 113.9 ± 2.7 | 135.1 ± 6.0$^{**}$ | 186.3 ± 4.5$^{**}$ |
| | (78.6 ± 1.4$^{**}$) | (100.0 ± 2.3) | (97.3 ± 1.2) | (96.7 ± 2.7) | (94.7 ± 0.8) |
| 14. Platycodon Root | 38.4 ± 1.2$^{**}$ | 100.0 ± 2.6 | 113.0 ± 1.9$^{*}$ | 130.6 ± 3.8$^{**}$ | 160.2 ± 3.2$^{**}$ |
| | (76.2 ± 1.4$^{**}$) | (100.0 ± 2.5) | (102.5 ± 1.4) | (105.7 ± 3.6) | (101.1 ± 1.0) |
| 15. Scutellaria Root | 39.9 ± 1.2$^{**}$ | 100.0 ± 4.1 | 105.4 ± 2.3 | 96.6 ± 1.7 | 86.5 ± 1.6$^{**}$ |
| | (77.5 ± 0.9$^{**}$) | (100.0 ± 1.3) | (96.0 ± 0.7) | (102.6 ± 1.8) | (106.0 ± 1.4) |
| 16. Glycyrrhiza | 49.8 ± 2.1$^{**}$ | 100.0 ± 6.1 | 114.3 ± 1.8$^{**}$ | 132.6 ± 2.5$^{**}$ | 122.6 ± 6.4$^{**}$ |
| | (76.2 ± 2.1$^{**}$) | (100.0 ± 2.2) | (101.9 ± 1.1) | (108.3 ± 2.5) | (75.2 ± 2.7$^{\#\#}$) |

Each value represents the mean ± S.E. (n = 4).
Significantly different from the control, $^{*}p < 0.05$, $^{**}p < 0.01$.
Values in parenthesis indicate percent of control of protein content in the homogenate.
Significantly different from the control, $^{\#}p < 0.05$, $^{\#\#}p < 0.01$.

**Table S5.** Effects of crude drug extracts of each component of bofutsushosan (BTS) on triglyceride contents in high-glucose-pretreated HepG2 cells

| Treatment | TG/protein (% of control) | | | |
|---|---|---|---|---|
| Conc. ($\mu$g/mL) | 0 | 30 | 100 | 300 |
| BTS ext. | 100.0 ± 1.9 | 96.3 ± 1.4 | 96.3 ± 2.2 | 90.2 ± 2.6** |
| | (100.0 ± 0.9) | (99.1 ± 1.3) | (100.4 ± 1.2) | (103.1 ± 1.1) |
| $H_2O$ extract | | | | |
| 1. Japanese Angelica Root | 100.0 ± 5.5 | 84.5 ± 2.0 | 92.4 ± 0.9 | 103.5 ± 3.2 |
| | (100.0 ± 2.4) | (99.4 ± 1.6) | (92.4 ± 1.4) | (86.0 ± 1.1##) |
| 2. Peony Root | 100.0 ± 1.1 | 95.3 ± 1.5** | 90.6 ± 1.7** | 85.1 ± 1.9** |
| | (100.0 ± 0.7) | (99.9 ± 1.4) | (102.5 ± 1.9) | (103.5 ± 1.6) |
| 3. Cnidium Rhizome | 100.0 ± 2.3 | 92.4 ± 0.9 | 92.2 ± 1.9 | 99.9 ± 2.0 |
| | (100.0 ± 0.9) | (105.7 ± 0.7) | (104.7 ± 1.6) | (102.9 ± 0.2) |
| 4. Gardenia Fruit | 100.0 ± 1.6 | 90.9 ± 2.3* | 89.2 ± 2.1** | 93.0 ± 2.6 |
| | (100.0 ± 0.7) | (101.1 ± 2.1) | (103.1 ± 0.8) | (101.2 ± 3.1) |
| 5. Forsythia Fruit | 100.0 ± 2.5 | 93.7 ± 2.6 | 82.7 ± 3.2** | 63.8 ± 0.8** |
| | (100.0 ± 1.3) | (102.7 ± 1.5) | (103.5 ± 1.8) | (109.0 ± 1.5) |
| 6. Mentha Herb | 100.0 ± 2.2 | 94.9 ± 2.2 | 90.0 ± 0.6* | 88.1 ± 4.2** |
| | (100.0 ± 0.7) | (101.8 ± 0.9) | (101.4 ± 0.6) | (102.1 ± 1.1) |
| 7. Ginger | 100.0 ± 1.8 | 90.1 ± 0.4** | 89.0 ± 1.6** | 93.0 ± 1.9* |
| | (100.0 ± 0.9) | (98.7 ± 1.0) | (100.2 ± 0.6) | (96.3 ± 1.0) |
| 8. Schizonepeta Spike | 100.0 ± 0.8 | 99.0 ± 0.6 | 89.3 ± 1.6** | 89.3 ± 1.6** |
| | (100.0 ± 0.7) | (100.0 ± 0.6) | (102.3 ± 0.4) | (100.2 ± 0.5) |
| 9. Saposhnikovia Root and Rhizome | 100.0 ± 2.0 | 88.3 ± 2.3** | 85.6 ± 1.1** | 90.4 ± 1.6** |
| | (100.0 ± 1.1) | (103.4 ± 0.7) | (103.4 ± 1.8) | (103.2 ± 0.3) |
| 10. Ephedra Herb | 100.0 ± 2.2 | 87.8 ± 3.1** | 82.2 ± 1.7** | 71.3 ± 1.8** |
| | (100.0 ± 2.2) | (105.3 ± 1.2) | (104.0 ± 0.6) | (105.6 ± 0.8) |
| 11. Rhubarb | 100.0 ± 1.2 | 89.9 ± 3.0** | 85.3 ± 1.0** | 64.6 ± 1.4** |
| | (100.0 ± 0.7) | (104.0 ± 1.9) | (106.7 ± 1.8) | (111.1 ± 0.9##) |
| 12. Sodium Sulfate | 100.0 ± 3.3 | 86.1 ± 1.7 | 91.1 ± 1.0 | 106.5 ± 3.4 |
| | (100.0 ± 1.9) | (107.3 ± 1.5) | (104.6 ± 1.2) | (98.0 ± 1.8) |
| 13. Atractylodes Rhizome | 100.0 ± 2.2 | 97.6 ± 1.3 | 96.9 ± 2.7 | 108.7 ± 2.7 |
| | (100.0 ± 0.9) | (99.2 ± 0.7) | (101.4 ± 1.0) | (100.7 ± 0.5) |
| 14. Platycodon Root | 100.0 ± 1.9 | 92.6 ± 0.9* | 92.8 ± 1.3* | 97.7 ± 2.1 |
| | (100.0 ± 1.3) | (103.5 ± 1.3) | (105.1 ± 2.7) | (95.1 ± 2.3) |
| 15. Scutellaria Root | 100.0 ± 1.2 | 91.8 ± 1.4** | 87.4 ± 0.6** | 87.2 ± 2.8** |
| | (100.0 ± 0.8) | (101.2 ± 0.9) | (106.1 ± 1.3) | (111.0 ± 1.9#) |
| 16. Glycyrrhiza | 100.0 ± 1.1 | 92.1 ± 2.8 | 91.6 ± 3.3* | 98.4 ± 3.3 |
| | (100.0 ± 0.6) | (103.0 ± 1.3) | (103.9 ± 1.0) | (104.8 ± 1.2) |

| Conc. ($\mu$g/mL) | 0 | 10 | 30 | 100 |
|---|---|---|---|---|
| MeOH extract | | | | |
| 1. Japanese Angelica Root | 100.0 ± 5.0 | 86.7 ± 2.8 | 87.7 ± 4.0 | 88.5 ± 1.6 |
| | (100.0 ± 3.1) | (107.8 ± 3.0) | (110.2 ± 3.6) | (102.6 ± 1.1) |
| 2. Peony Root | 100.0 ± 1.6 | 94.1 ± 3.6 | 91.1 ± 0.6* | 83.7 ± 2.6** |
| | (100.0 ± 1.6) | (102.5 ± 2.2) | (103.0 ± 1.2) | (104.1 ± 2.4) |
| 3. Cnidium Rhizome | 100.0 ± 6.9 | 69.0 ± 5.1** | 66.4 ± 5.1** | 83.3 ± 4.3** |
| | (100.0 ± 1.3) | (104.4 ± 0.5) | (108.4 ± 0.9) | (106.8 ± 1.0) |
| 4. Gardenia Fruit | 100.0 ± 2.3 | 91.0 ± 1.8* | 87.8 ± 3.0** | 82.5 ± 2.5** |
| | (100.0 ± 1.3) | (108.5 ± 2.6) | (109.5 ± 3.1) | (114.2 ± 4.5##) |
| 5. Forsythia Fruit | 100.0 ± 2.2 | 85.6 ± 3.1** | 66.2 ± 2.0** | 47.5 ± 1.6** |
| | (100.0 ± 2.0) | (104.1 ± 2.6) | (107.8 ± 2.0) | (104.2 ± 3.1) |
| 6. Mentha Herb | 100.0 ± 2.1 | 87.0 ± 2.8** | 88.1 ± 1.7** | 81.5 ± 0.8** |
| | (100.0 ± 1.2) | (105.5 ± 1.2) | (104.0 ± 1.7) | (106.5 ± 1.0) |
| 7. Ginger | 100.0 ± 1.6 | 95.2 ± 3.7 | 95.3 ± 2.8 | 86.5 ± 1.9** |
| | (100.0 ± 1.1) | (98.9 ± 2.0) | (99.6 ± 1.0) | (102.4 ± 1.2) |
| 8. Schizonepeta Spike | 100.0 ± 2.9 | 95.8 ± 2.3 | 91.5 ± 2.8 | 77.6 ± 2.0** |
| | (100.0 ± 1.5) | (100.3 ± 1.9) | (105.6 ± 1.7) | (113.4 ± 0.7##) |
| 9. Saposhnikovia Root and Rhizome | 100.0 ± 2.3 | 93.0 ± 3.6 | 90.8 ± 1.9 | 85.9 ± 1.2** |
| | (100.0 ± 0.9) | (101.6 ± 1.5) | (102.1 ± 1.5) | (104.2 ± 0.7) |
| 10. Ephedra Herb | 100.0 ± 3.3 | 108.8 ± 3.5 | 102.7 ± 6.9 | 92.2 ± 3.2 |
| | (100.0 ± 1.3) | (94.6 ± 0.6) | (102.7 ± 3.5) | (105.2 ± 0.9) |
| 11. Rhubarb | 100.0 ± 1.2 | 93.1 ± 1.9 | 87.2 ± 4.3** | 79.1 ± 2.9** |
| | (100.0 ± 0.9) | (107.1 ± 2.6) | (107.0 ± 2.2) | (118.3 ± 1.6##) |
| 13. Atractylodes Rhizome | 100.0 ± 1.7 | 101.6 ± 1.9 | 107.4 ± 0.7 | 119.5 ± 3.6 |
| | (100.0 ± 0.9) | (100.6 ± 1.5) | (98.1 ± 1.8) | (99.7 ± 1.1) |
| 14. Platycodon Root | 100.0 ± 1.7 | 95.5 ± 2.0 | 97.6 ± 2.6 | 96.3 ± 1.6 |
| | (100.0 ± 1.0) | (104.2 ± 0.5) | (105.5 ± 0.7) | (104.1 ± 0.7) |
| 15. Scutellaria Root | 100.0 ± 2.6 | 92.9 ± 0.5 | 93.8 ± 3.7 | 83.0 ± 2.8** |
| | (100.0 ± 1.4) | (103.1 ± 1.2) | (103.1 ± 2.4) | (108.3 ± 1.0) |
| 16. Glycyrrhiza | 100.0 ± 2.2 | 92.9 ± 0.6 | 88.3 ± 3.2** | 52.5 ± 1.0** |
| | (100.0 ± 0.7) | (103.9 ± 0.4) | (107.0 ± 1.7) | (109.9 ± 0.4##) |

Each value represents the mean ± S.E. (n = 4).
Significantly different from the control, $^{*}p < 0.05$, $^{**}p < 0.01$.
Values in parenthesis indicate percent of control of protein content in the homogenate.
Significantly different from the control, $^{\#}p < 0.05$, $^{\#\#}p < 0.01$.
